# MRI VS. FDG-PET for diagnosis of response to neoadjuvant therapy in patients with locally advanced rectal cancer

**DOI:** 10.3389/fonc.2023.1031581

**Published:** 2023-01-18

**Authors:** Peng Fei Gao, Na Lu, Wen Liu

**Affiliations:** ^1^ Department of Traditional Chinese medicine, Jinshan Hospital, Fudan University, Shanghai, China; ^2^ Department of Radiology, Huashan Hospital North, Fudan University, Shanghai, China; ^3^ Department of Radiology, Jinshan Hospital, Fudan University, Shanghai, China

**Keywords:** rectal cancer, diagnostic value, MRI, FDG-PET, FDG-PET/CT, neoadjuvant therapy, response

## Abstract

**Aim:**

In this study, we aimed to compare the diagnostic values of MRI and FDG-PET for the prediction of the response to neoadjuvant chemoradiotherapy (NACT) of patients with locally advanced Rectal cancer (RC).

**Methods:**

Electronic databases, including PubMed, Embase, and the Cochrane library, were systematically searched through December 2021 for studies that investigated the diagnostic value of MRI and FDG-PET in the prediction of the response of patients with locally advanced RC to NACT. The quality of the included studies was assessed using QUADAS. The pooled sensitivity, specificity, positive and negative likelihood ratio (PLR and NLR), and the area under the ROC (AUC) of MRI and FDG-PET were calculated using a bivariate generalized linear mixed model, random-effects model, and hierarchical regression.

**Results:**

A total number of 74 studies with recruited 4,105 locally advanced RC patients were included in this analysis. The pooled sensitivity, specificity, PLR, NLR, and AUC for MRI were 0.83 (95% CI: 0.77–0.88), 0.85 (95% CI: 0.79–0.89), 5.50 (95% CI: 4.11-7.35), 0.20 (95% CI: 0.14–0.27), and 0.91 (95% CI: 0.88–0.93), respectively. The summary sensitivity, specificity, PLR, NLR and AUC for FDG-PET were 0.81 (95% CI: 0.77-0.85), 0.75 (95% CI: 0.70–0.80), 3.29 (95% CI: 2.64–4.10), 0.25 (95% CI: 0.20–0.31), and 0.85 (95% CI: 0.82–0.88), respectively. Moreover, there were no significant differences between MRI and FDG-PET in sensitivity (*P* = 0.565), and NLR (*P* = 0.268), while the specificity (*P* = 0.006), PLR (*P* = 0.006), and AUC (*P* = 0.003) of MRI was higher than FDG-PET.

**Conclusions:**

MRI might superior than FGD-PET for the prediction of the response of patients with locally advanced RC to NACT.

## Introduction

Rectal cancer (RC) as is a common malignant tumor, with nearly 39,910 new cases in US annually (1, 2). Currently, surgical resection is the main curative method for patients with early-stage RC, whereas nearly 55% of RC cases are diagnosed at stage II or higher, when additional treatment strategies are needed (3, 4). Neoadjuvant chemoradiotherapy (NACT), total mesorectal excision, and postoperative chemotherapy are standard treatment strategies in patients with locally advanced RC (5, 6). Earlier studies showed that NACT improved locoregional control with significant pathologic complete response (pCR), which was defined as the absence of viable tumor cells established by pathologic examination (7–10). The tumor responses to NACT ranged from sustained tumor progression to complete remission, and adjuvant postoperative therapy could affect by the heterogeneity of patients’ tumor response to NACT. Previous evidence indicated that surgery could be omitted in patients with pCR to NACT, in which the watch-and-wait strategy was associated with better prognosis (11, 12). Therefore, the accurate assessment of the response to NACT could contribute to more effective clinical care aimed at personalized treatment strategy in patients with locally advanced RC.

Recent studies established the role of imaging modalities, including fluorine-18 fluorodeoxyglucose-positron emission tomography (^18^FDG-PET), irrespective whether combined with computed tomography (CT) or MRI in the prediction of the response to NACT (13, 14). The apparent diffusion coefficient (ADC), measured by MRI, could facilitate tumor cellularity and cell membrane integrity which are sensitive to intratumoral changes induced by NACT. MRI was found to have a relatively better predictive value for the tumor response during and after neoadjuvant therapy (15). FDG-PET has been widely used for the diagnosis of recurrent or metastatic colorectal cancer (CRC), with a detection accuracy rate for pelvic recurrence within 74%–96% (16–18). ^18^FDG-PET combined with CT (FDG-PET/CT) showed an even higher accuracy rate for diagnosing locally recurrent and metastatic CRC (19, 20). Several studies revealed that FDG-PET predicted successfully the response to NACT, while the predictive value between MRI and FDG-PET for the response to NACT in locally advanced RC patients remains controversial. Therefore, here, we performed a meta-analysis focused on indirect comparisons between the diagnostic values of MRI and FDG-PET for the assessment of the response to NACT.

## Materials and methods

### Data sources, search strategy, and selection criteria

This review was conducted and reported according to the Preferred Reporting Items for Systematic Reviews and Meta-Analysis Statement issued in 2009 (21). Studies that had investigated the diagnostic value of MRI or FDG-PET for the assessment of the response to NACT in patients with locally advanced RC were eligible for inclusion in this analysis, with no restrictions placed on the publication language and status. The PubMed, Embase, and Cochrane Library electronic databases were searched for articles published through December 2021. The following search terns were used: “Magnetic Resonance Imaging” OR “Positron-Emission Tomography” OR “computed tomography” AND “rectal cancer” AND “preoperative” OR “neoadjuvant”. The details of searching strategy in PubMed are specified in Supplemental 1. We also conducted manual searches of the reference lists of all relevant original and review articles to identify additional eligible studies.

The literature search and study selection were independently performed by two authors using a standardized approach. Any inconsistencies between authors were settled by consultation and discussion with an additional author until a consensus was reached. The following inclusion criteria were applied: (1) Study design: prospective or retrospective design; (2) Participants: all patients were diagnosed with locally advanced RC by pathologic examination; (3) Diagnostic tool: MRI, FDG-PET, or FDG-PET/CT; (4) Gold reference: tumor response diagnosed using the postoperative histological results; and (5) Outcomes: true and false positive, true and false negative, or data could be transformed into the aforementioned information data.

### Data collection and quality assessment

The data collection and quality assessment were conducted by two authors, and the information collected was examined and adjudicated by an additional author. The data collected included the first author’s surname, publication year, country, study design, sample size, median or mean age, number of men and women included, preoperative regimen, diagnostic tool, responders and non-responders, true and false positive, and true and false negative. The quality of the included studies was assessed using QUADAS, based on 14 items; “yes”, “no”, or “unclear” were the possible answers to each question/item. A study that had collected 12 or more “yes” answers was regarded to have high quality, and those that received 10–12 “yes” answers for were considered to be of moderate quality.

### Statistical analysis

The sensitivity, specificity, positive likelihood ratio (PLR), negative likelihood ratio (NLR), and the area under the receiver operating characteristic curves (AUC) with corresponding 95% confidence intervals (CIs) were calculated based on true positive, false positive, false negative, and true negative results in each individual study before data pooling. Then, the pooled sensitivity, specificity, PLR, NLR, and AUC for each diagnostic tool were calculated using a bivariate generalized linear mixed model, random-effects model, and hierarchical regression (22–24). Heterogeneity across the included studies was evaluated by *I^2^* and Q statistic; *P* < 0.10 was considered to indicate significant heterogeneity (25). Subgroup analyses for sensitivity, specificity, PLR, NLR, and AUC were conducted based on the study design (retrospective or prospective), sample size (>50 and <50), and mean age (>60.0 and <60.0). The ratio of between the MRI and FDG-PET diagnostic parameters in the subgroups were calculated for indirect comparison of between the MRI and FDG-PET diagnostic values (26). The publication biases for CT and FDG-PET were assessed using funnel plots and Deeks’ asymmetry tests (27). The *P-*value for all pooled analyses were two-sided; *P* < 0.05 was considered to indicate statistically significant differences. Stata software (version 10.0; Stata Corporation, College Station, TX, USA) was employed to conduct all statistical analyses.

## Results

### Literature search

**Figure 1:**
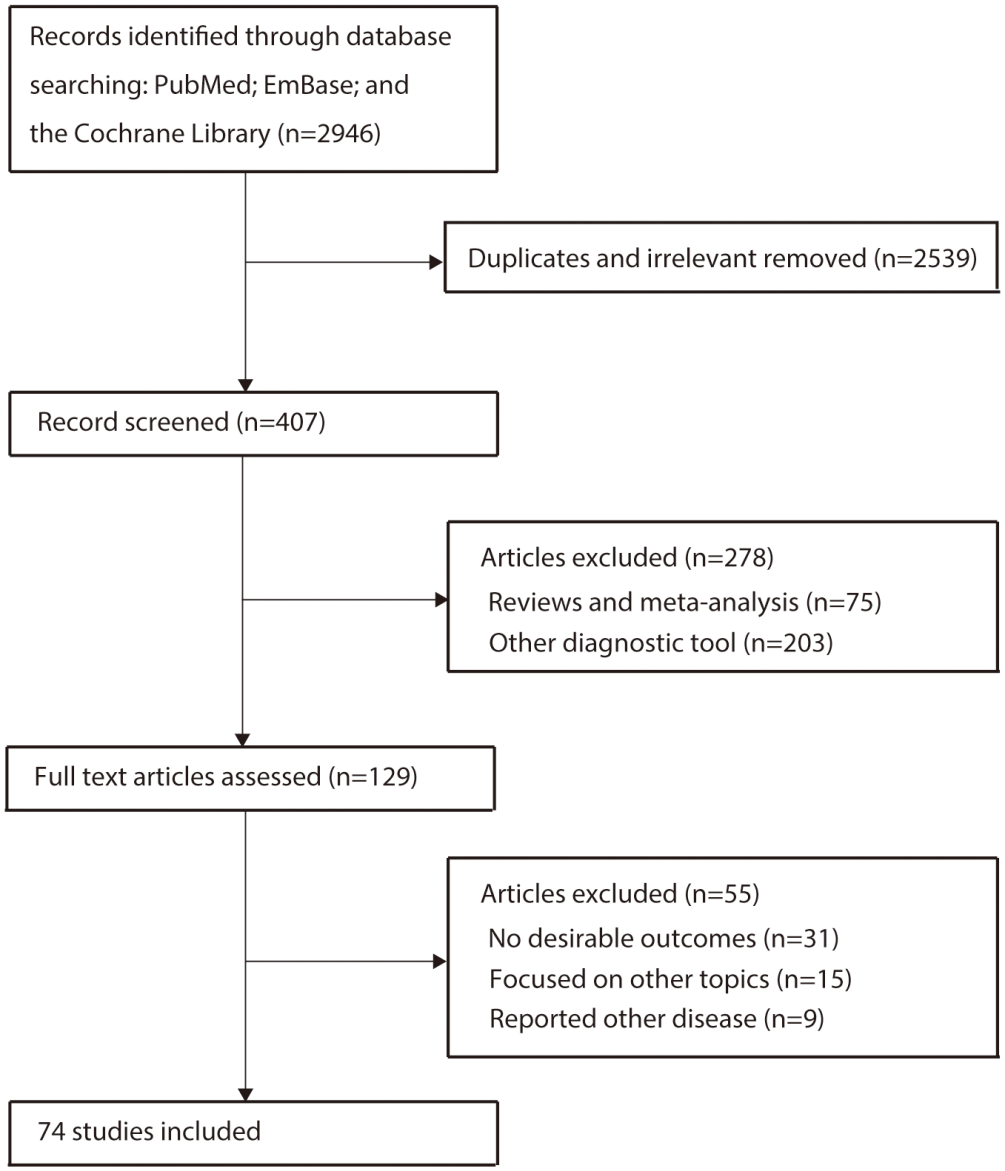
Flow diagram of the study selection process.

The results of the study-selection process are depicted in **Figure 1**. We initially identified 2946 potentially eligible articles after the original electronic search. Of these, 2539 articles were excluded during an initial review of the titles. Abstracts assessment for 407 articles, and 278 studies were excluded due to the use of other diagnostic tools and review designs. The remaining 129 studies were subjected to further tests to identify any other potential studies eligible for inclusion, and 74 of them satisfied the inclusion criteria and were ultimately included in the quantitative analysis (28–101). A manual search of the reference lists contained within these studies did not yield any new eligible studies. The general characteristics of the included studies are presented in **Table 1**.

**Table 1:** The baseline characteristics of included studies.

Study and publication year	Country	Study design	Sample size	Age (years)	No of men and women	Preoperative regimen	Diagnostic tool	Responders and non-responders	Study quality
Amthauer 2004 (28)	Germany	Pro	20	53.1	14/6	RC	FDG-PET	Res: 13; NR: 7	Moderate
Capirci 2004 (29)	Italy	Retro	81	63.9	53/28	RC	FDG-PET	Res: 49; NR: 32	Moderate
Denecke 2005 (30)	Germany	Pro	23	53.0	16/7	RC	FDG-PET	Res: 13; NR: 10	Moderate
Cascini 2006 (31)	Italy	Pro	33	58.0	20/13	RC	FDG-PET	Res: 18; NR: 15	Moderate
Melton 2007 (32)	USA	Retro	21	61.0	13/8	RC	FDG-PET/CT	Res: 14; NR: 7	Moderate
Kristiansen 2008 (33)	Denmark	Retro	30	63.0	16/14	RC	FDG-PET/CT	Res: 14; NR: 16	Moderate
Capirci 2009 (34)	Italy	Pro	81	58.0	58/23	RC	FDG-PET/CT	Res: 40; NR: 41	High
Rosenberg 2009 (35)	Germany	Pro	30	61.0	20/10	RC	FDG-PET/CT	Res: 19; NR: 10	Moderate
Palma 2010 (36)	Spain	Pro	50	60.0	37/13	RC	FDG-PET/CT	Res: 20; NR: 30	Moderate
Lambrecht 2010 (37)	Belgium	Pro	22	59.8	17/5	RC	FDG-PET/CT	Res: 6; NR: 16	Moderate
Martoni 2011 (38)	Italy	Pro	80	65.0	55/25	RC	FDG-PET/CT	CR: 16; IR: 20; NR: 48	High
Hur 2011 (39)	Korea	Pro	37	59.0	25/12	RC	FDG-PET	Res: 25; NR: 12	Moderate
Yoon 2011 (40)	Korea	Pro	72	66.0	56/16	RC	FDG-PET/CT	Res: 43; NR: 29	Moderate
Kim 2011 (41)	Korea	Pro	34	58.1	24/10	RC	MRI	Res: 16; NR: 18	Moderate
Kim 2011 (42)	Korea	Retro	76	60.0	49/27	RC	MRI	CR: 11, nearly CR: 14; MR: 51	Moderate
Herrmann 2011 (43)	Germany	Pro	28	61.0	20/8	RC	FDG-PET/CT	Res: 20; NR: 8	Moderate
Guerra 2011 (44)	Italy	Pro	31	67.0	23/8	RC	FDG-PET/CT	Res: 22; NR: 9	Moderate
Everaert 2011 (45)	Belgium	Pro	45	65.4	34/11	R	FDG-PET	Res: 20; NR: 25	Moderate
Curvo-Semedo 2011 (46)	Netherlands	Retro	50	71.5	36/14	RC	MRI	CR: 14; IR: 36	Moderate
Song 2012 (47)	Korea	Retro	50	56.0	39/11	RC	MRI; FDG-PET/CT	CR: 6; near CR: 13; MR: 31	Moderate
Ippolito 2012 (48)	Italy	Pro	30	66.0	21/9	RC	MRI; FDG-PET/CT	Res: 21; NR: 9	Moderate
Perez 2012 (49)	Brazil	Pro	99	60.3	47/52	RC	FDG-PET/CT	CR: 18; IR: 81	High
Lambrecht 2012 (50)	Belgium	Retro	20	60.0	16/4	RC	MRI	CR: 6; NR: 14	Moderate
Jung 2012 (51)	Korea	Retro	35	62.0	29/6	RC	MRI	Res: 23; NR: 12	Moderate
Janssen 2012 (52)	Netherlands	Pro	51	NA	NA	RC	FDG-PET/CT	Res: 17; NR: 29	Moderate
Huh 2012 (53)	Korea	Pro	50	64.0	38/12	RC	FDG-PET/CT	Res: 32; NR: 18	Moderate
Chennupati 2012 (54)	USA	Retro	35	NA	NA	RC	FDG-PET/CT	CR: 6; near-CR: 8; NR: 21	Moderate
Barbaro 2012 (55)	Italy	Pro	62	64.0	43/19	RC	MRI	Res: 37; NR: 25	High
Guillem 2013 (56)	USA	Pro	121	60.0	76/45	RC	FDG-PET	CR: 26; IR: 95	High
Hatt 2013 (57)	France	Retro	28	67.0	18/10	RC	FDG-PET	Res: 12; NR: 16	Moderate
Murcia Duréndez 2013 (58)	Spain	Pro	41	66.0	25/16	RC	FDG-PET/CT	Res: 14; NR: 27	Moderate
Calvo 2013 (59)	Spain	Pro	38	62.0	27/11	RC	FDG-PET/CT	Res: 19; NR: 19	Moderate
Sun 2013 (60)	China	Pro	53	53.0	44/9	RC	FDG-PET/CT	Res: 21; NR: 32	Moderate
Genovesi 2013 (61)	Italy	Pro	28	68.3	17/11	RC	MRI	Res: 10; NR: 18	Moderate
Park 2014 (62)	Korea	Retro	88	59.2	64/24	RC	FDG-PET/CT	CR: 17; non-CR: 71	Moderate
Niccoli-Asabella 2014 (63)	Italy	Pro	56	62.3	38/18	RC	FDG-PET/CT	Res: 23; NR: 33	High
Cai 2014 (64)	China	Retro	65	56.0	52/13	RC	MRI	Res: 43; NR: 22	Moderate
Aiba 2014 (65)	Japan	Retro	40	56.0	32/8	C	MRI; FDG-PET/CT	Res: 16; NR: 24	High
Doi 2015 (66)	Japan	Pro	16	62.5	13/3	RC	MRI	Res: 9; NR: 7	Moderate
Blažić 2015 (67)	Serbia	Pro	58	61.3	38/20	RC	MRI	Res: 19; NR: 39	Moderate
Martens 2015 (68)	Netherlands	Retro	146	64.6	90/56	RC	MRI	CR: 29; non-CR: 117	High
Petrillo 2015 (69)	Italy	Pro	29	62.0	NA	RC	MRI	Res: 14; NR: 15	Moderate
Choi 2015 (70)	Korea	Retro	86	64.3	58/28	RC	MRI	CR: 16; non-CR: 70	High
Leccisotti 2015 (71)	Italy	Pro	126	65.0	79/47	RC	FDG-PET/CT	CR: 31; non-CR: 95	High
Tong 2015 (72)	China	Pro	38	52.0	25/13	RC	MRI	CR: 12; non-CR: 26	Moderate
Martens 2015 (73)	Netherlands	Pro	30	66.0	23/7	RC	MRI	Res: 13; NR: 17	Moderate
Altini 2015 (74)	Italy	Pro	68	63.0	41/27	RC	FDG-PET/CT	Res: 25; NR: 43	Moderate
Lambregts 2015 (75)	Netherlands	Retro	112	67.0	76/36	RC	MRI	CR: 20; non-CR: 92	Moderate
Koo 2016 (76)	Korea	Retro	103	66.0	78/25	RC	FDG-PET/CT	CR: 22; non-CR: 81	High
Travaini 2016 (77)	Italy	Pro	41	61.0	26/15	RC	FDG-PET/CT	Res: 23; NR: 18	Moderate
Li 2016 (78)	China	Pro	64	53.0	49/15	RC	FDG-PET/CT	Res: 31; NR: 33	Moderate
De Cecco 2016 (79)	Italy	Pro	12	63.2	4/8	RC	MRI	Res: 9; NR: 3	Moderate
Chen 2016 (80)	China	Retro	100	55.0	68/32	RC	MRI	CR: 50; non-CR: 50	Moderate
Iannicelli 2016 (81)	Italy	Pro	34	65.0	19/15	RC	MRI	Res: 11; NR: 23	Moderate
Sathyakumar 2016 (82)	India	Pro	64	49.5	48/16	RC	MRI	CR: 11; non-CR: 53	High
Jacobs 2016 (83)	Netherlands	Pro	22	62.9	16/6	RC	MRI	Res: 9; NR: 13	Moderate
Petrillo 2017 (84)	Italy	Retro	35	67.0	27/8	R	MRI	Res: 16; NR: 19	Moderate
Bassaneze 2017 (85)	Brazil	Retro	33	59.6	18/15	RC	MRI	CR: 7; non-CR: 26	Moderate
De Felice 2017 (86)	Italy	Pro	37	62.0	28/9	RC	MRI	CR: 11; non-CR: 26	Moderate
Zhu 2017 (87)	China	Pro	98	57.5	64/34	RC	MRI	CR: 19; non-CR: 79	High
Yu 2017 (88)	China	Retro	41	NA	25/16	RC	MRI	Res: 17; NR: 24	Moderate
Petrillo 2018 (89)	Italy	Pro	88	66.0	62/26	RC	MRI	Res: 52; NR: 36	Moderate
Fusco 2018 (90)	Italy	Retro	34	67.0	26/8	R	MRI	Res: 15; NR: 19	Moderate
Murata 2018 (91)	Japan	Retro	36	66.0	27/9	RC	MRI; FDG-PET/CT	CR: 10; non-CR: 26	Moderate
Liu 2018 (92)	China	Pro	124	59.0	75/49	RC	MRI	CR: 20; non-CR: 104	Moderate
Aker 2018 (93)	UK	Retro	103	NA	NA	RC	MRI	CR: 20; non-CR: 83	Moderate
Horvat 2018 (94)	Brazil	Retro	114	55.0	67/47	RC	MRI	CR: 21; non-CR: 93	High
Pizzi 2018 (95)	Italy	Pro	43	67.4	22/21	RC	MRI	CR: 21; non-CR: 22	High
Nahas 2019 (96)	Brazil	Retro	95	62.9	58/37	RC	MRI	CR: 20; non-CR: 75	Moderate
Giannini 2019 (97)	Italy	Retro	52	68.0	35/17	RC	MRI; FDG-PET	Res: 22; NR: 30	Moderate
Palmisano 2020 (98)	Italy	Pro	43	61.0	27/16	RC	MRI	Res: 33; NR: 10	High
Bae 2020 (99)	Korea	Retro	38	60.0	17/21	RC	MRI	CR: 26; non-CR: 12	Moderate
López-López 2021 (100)	Spain	Pro	68	63.4	36/32	RC	FDG-PET/CT	CR: 15; non-CR: 53	High
Uemura 2021 (101)	Japan	Retro	40	68.5	26/14	RC	MRI	Res: 17; NR: 23	Moderate

*C, chemotherapy; CR, complete responder; IR, incomplete responder; MR, moderate or minimal responder; NR, non-responder; Pro, prospective; R, radiotherapy; RC, radiochemotherapy; Res, responders; Retro, retrospective.

### Study characteristics

Seventy-four studies with a total number of 4,105 patients with locally advanced RC were included in this analysis. Forty-five studies were designed as prospective, whereas the remaining 29 studies were designed as retrospective. The mean age of the patients was 49.5–71.5 years; 12–146 individuals were included in each of the included studies. Seventy studies employed radiochemotherapy as preoperative regimen, whereas radiotherapy or chemotherapy was used as preoperative regimen in the remaining four studies. The predictive value of MRI for the response to NACT was established in 41 studies, the predictive value of FDG-PET in 9 studies, and the predictive value of FDG-PET/CT in 29 studies. Seventeen of included studies were of high quality, whereas the remaining 57 studies were of moderate quality.

### MRI

**Figure 2:**
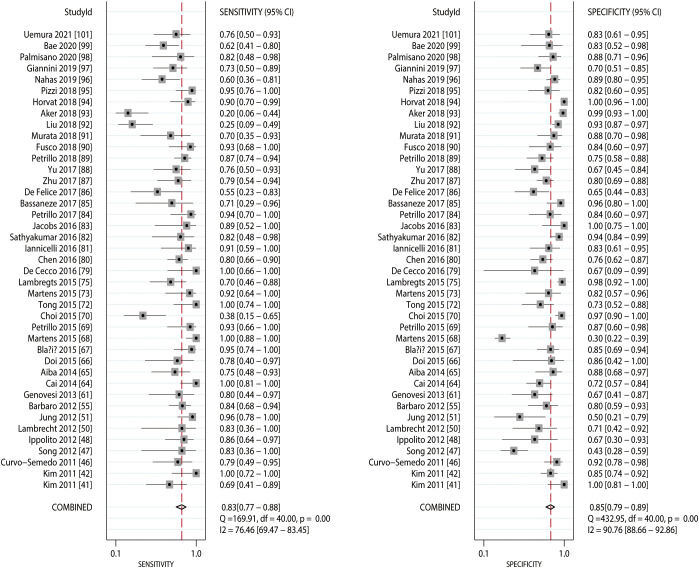
Pooled sensitivity and specificity of MRI.

**Figure 3:**
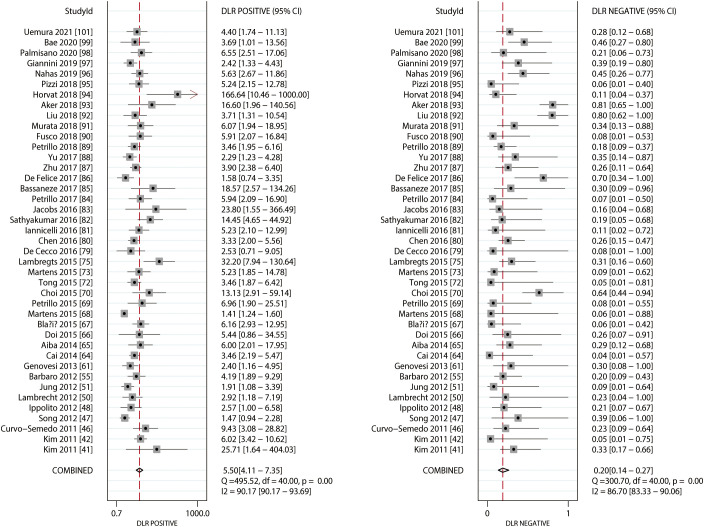
Pooled PLR and NLR of MRI.

**Figure 4:**
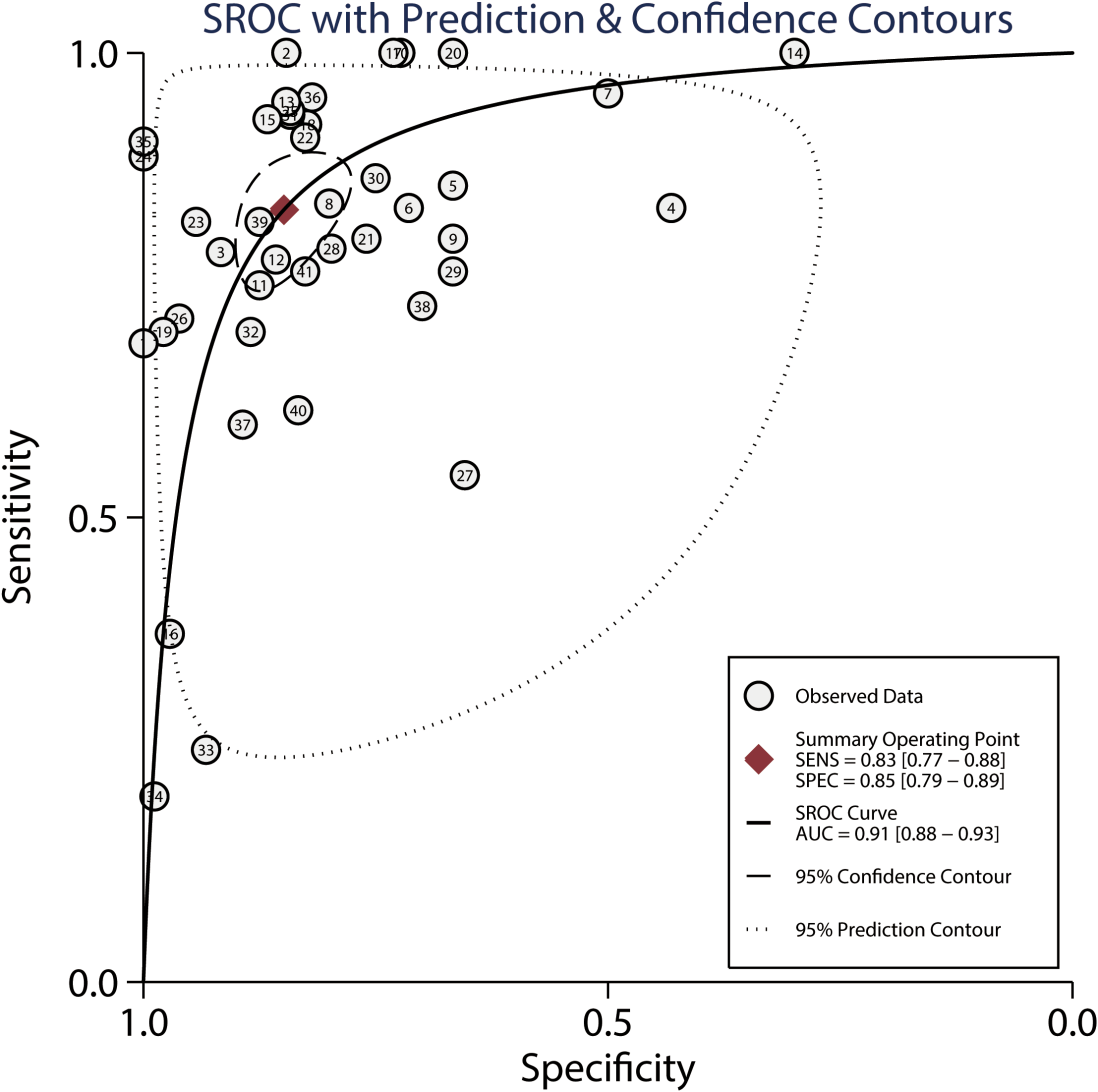
Summarized ROC curve and AUC for MRI.

**Figure 5:**
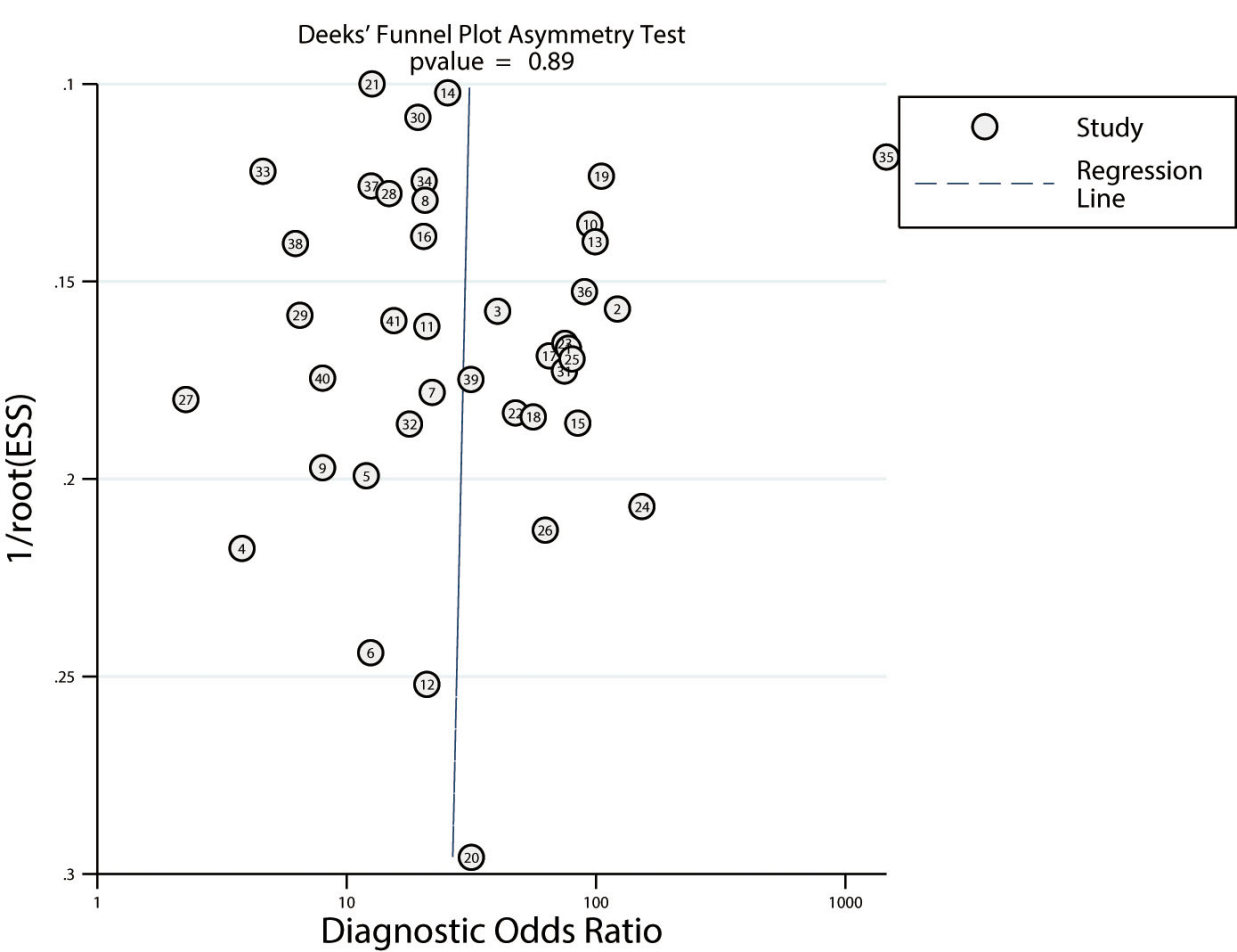
Publication biases for MRI.

The sensitivity and specificity are presented in **Figure 2**. The pooled sensitivity and specificity of MRI for predicting the response to NACT were 0.83 (95% CI: 0.77–0.88) and 0.85 (95% CI: 0.79–0.89), respectively. Substantial heterogeneity in the sensitivity (*I^2 =^* 76.46%; *P*<0.01) and specificity (*I^2 =^* 90.76%; *P*<0.01) of the included studies was observed. Moreover, the summarized PLR and NLR of MRI for predicting the response to NACT were 5.50 (95% CI: 4.11–7.35) and 0.20 (95% CI: 0.14–0.27), respectively (**Figure 3**), with significant heterogeneity in PLR (*I^2 =^* 90.17%; *P*<0.01) and NLR (*I^2 =^* 86.70%; *P*<0.01) across the included studies. In addition, the summarized AUC of MRI for predicting the response to NACT was 0.91 (95% CI: 0.88–0.93; **Figure 4**). Finally, there was no significant publication bias for MRI (*P* = 0.89; **Figure 5**).

### FDG-PET

**Figure 6:**
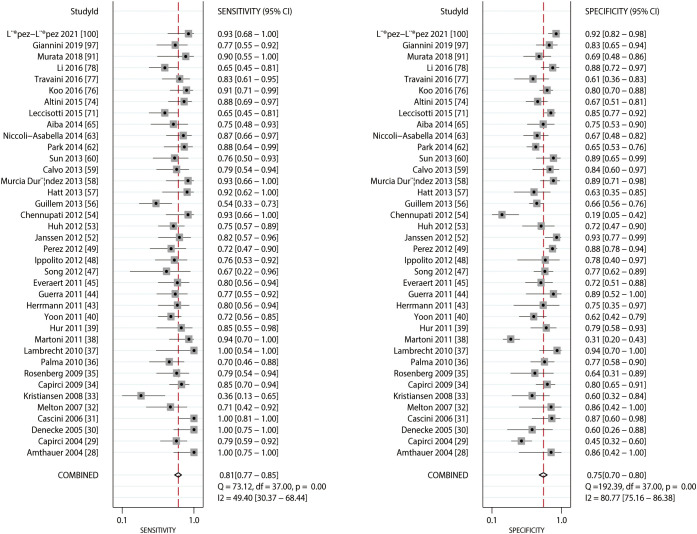
Pooled sensitivity and specificity of FDG-PET and FDG-PET/CT.

**Figure 7:**
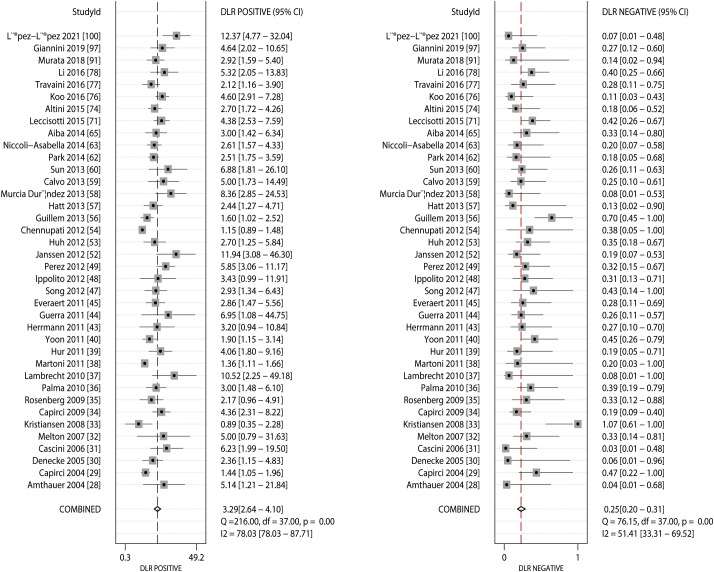
Pooled PLR and NLR of FDG-PET and FDG-PET/CT.

**Figure 8:**
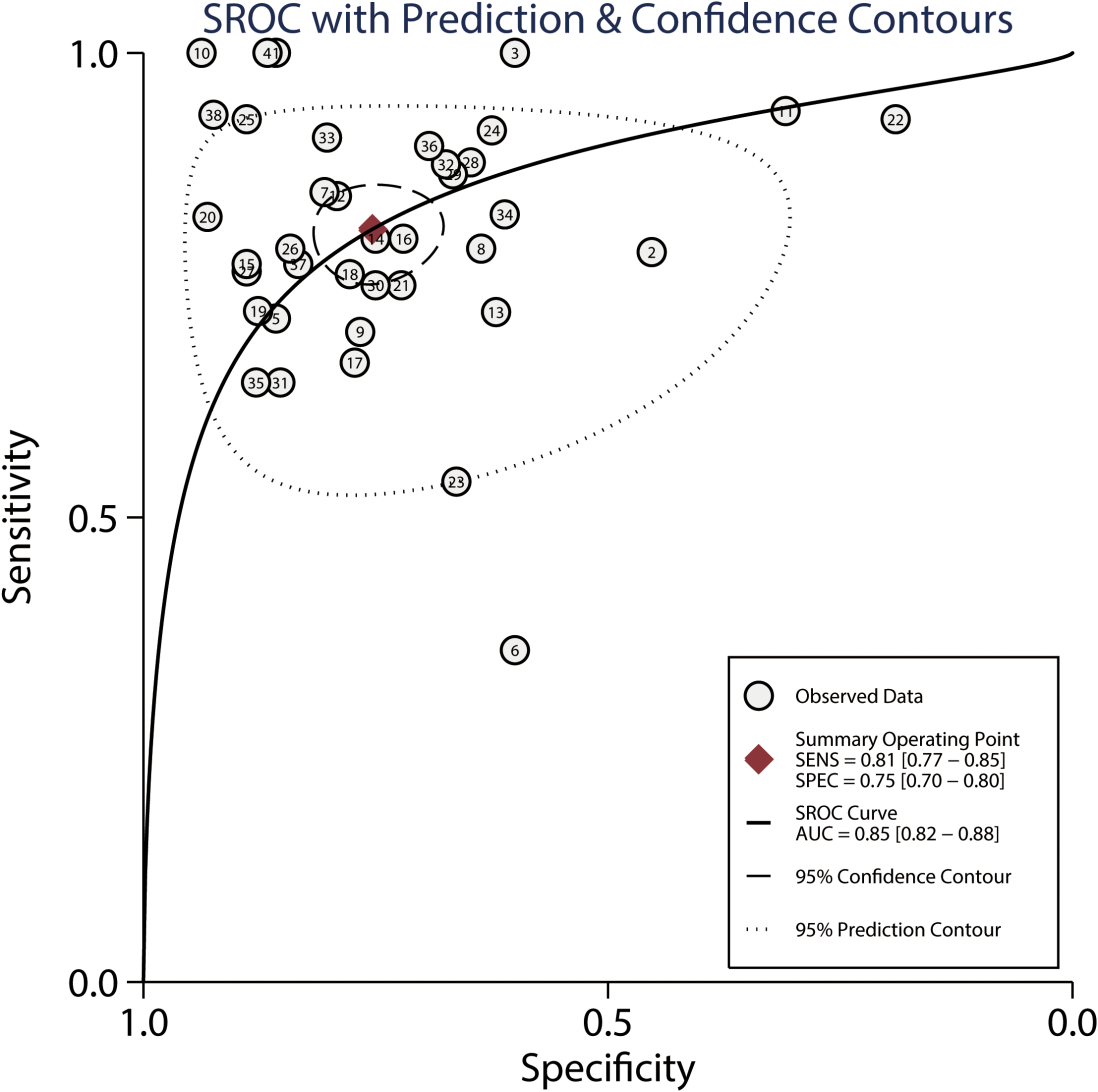
Summarized ROC curve and AUC for FDG-PET and FDG-PET/CT.

**Figure 9:**
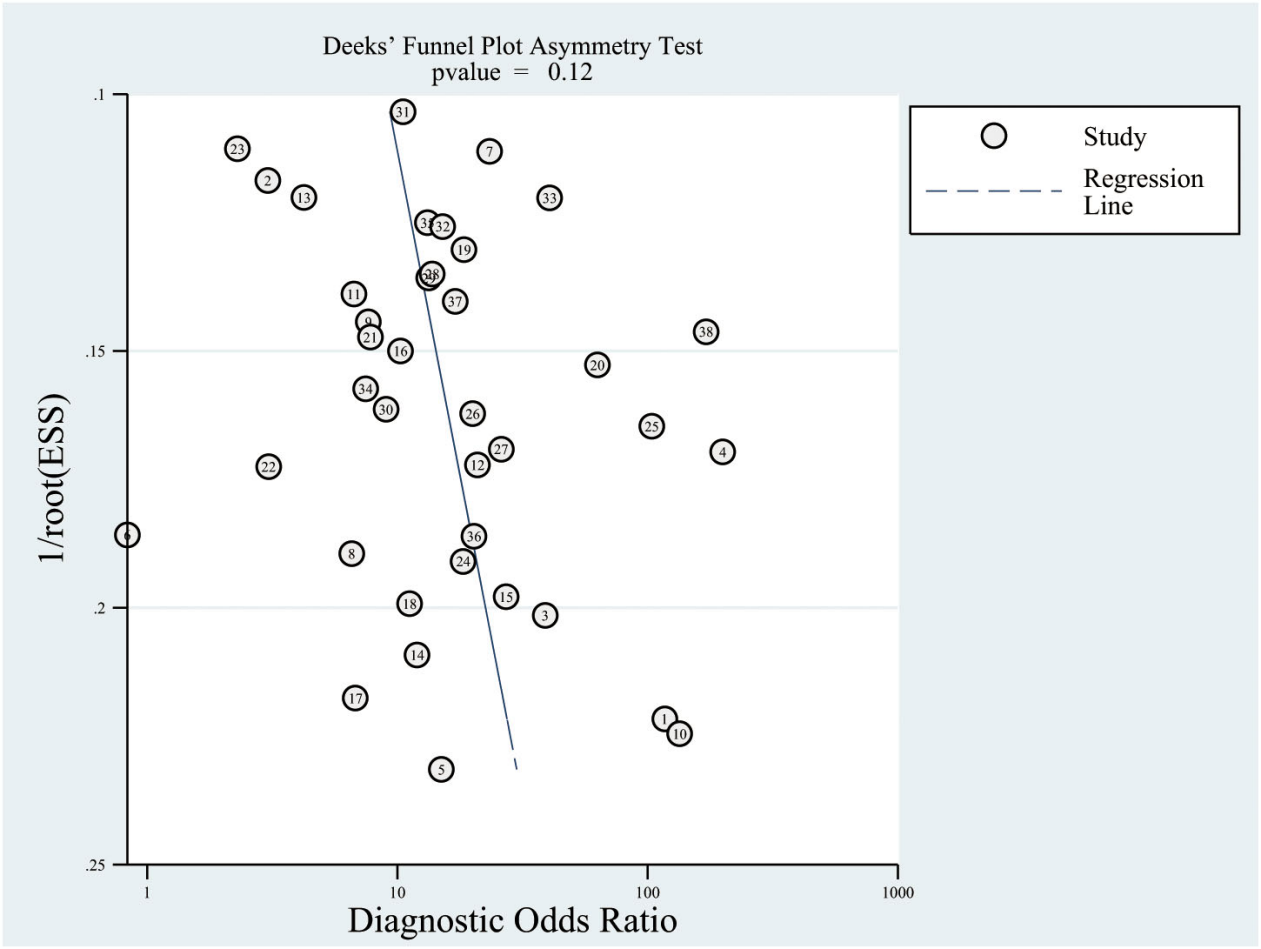
Publication biases for FDG-PET and FDG-PET/CT.

The summarized sensitivity and specificity are illustrated in **Figure 6**. The pooled sensitivity and specificity of FDG-PET for predicting the response to NACT were 0.81 (95% CI: 0.77–0.85) and 0.75 (95% CI: 0.70–0.80), respectively. Significant heterogeneity was detected in the sensitivity (*I^2 =^* 49.40%; *P*<0.01) and specificity (*I^2 =^* 80.77%; *P*<0.01) of FDG-PET. Moreover, the pooled PLR and NLR of FDG-PET for predicting the response to NACT were 3.29 (95% CI: 2.64–4.10) and 0.25 (95% CI: 0.20–0.31) respectively, with substantial heterogeneity for PLR (*I^2 =^* 78.03%; *P*<0.01) and NLR (*I^2 =^* 51.41%; *P*<0.01) across the included studies (**Figure 7**). In addition, the summary AUC of FDG-PET was 0.85 (95% CI: 0.82–0.88; **Figure 8**). Finally, no significant publication bias was observed in FDG-PET (*P* = 0.12; **Figure 9**).

### Indirect comparison of MRI and FDG-PET

The indirect comparison of the predictive values of MRI and FDG-PET for the response to NACT were calculated, the results of which suggested no significant differences between MRI and FDG-PET or FDG-PET/CT for the response to neoadjuvant chemoradiotherapy in patients with locally advanced RC, in terms of sensitivity (ratio: 1.02; 95% CI: 0.94–1.11; *P* = 0.565), and NLR (ratio: 0.80; 95% CI: 0.54–1.19; *P* = 0.268). Moreover, we noted the specificity (ratio: 1.13; 95% CI: 1.04–1.24; *P* = 0.006), PLR (ratio: 1.67; 95% CI: 1.16–2.41; *P* = 0.006), and AUC (ratio: 1.07; 95% CI: 1.02–1.12; *P* = 0.003).

### Meta-regression and subgroup analyses

The results of our meta-regression analyses showed that the sample size and mean age affected the sensitivity of MRI, whereas the study design did not affect MRI sensitivity. Moreover, the study design, sample size, and mean age did not affect the specificity of MRI. No bias was established in the sensitivity and specificity of FDG-PET exerted by study design, sample size, and mean age (Supplemental 2). The results of the subgroup analyses regarding the sensitivity, specificity, PLR, NLR, and AUC of MRI and FDG-PET are presented in **Table 2**. We noted a higher specificity in patients that had received MRI than in those subjected to FDG-PET if pooled retrospective studies (*P* = 0.010), at a sample size > 50 (*P* = 0.046). Furthermore, MRI had a higher PLR than FDG-PET when the pooled study was designed as retrospective (*P* = 0.003), with a sample size > 50 (*P* = 0.027) and a mean age of the patients > 60.0 years (*P* = 0.013). Finally, MRI was associated with lower NLR than FDG-PET if mean age of the patients > 60.0 years (*P* = 0.033).

**Table 2:** Subgroup analyses for diagnostic parameters.

Parameters	Variable	Group	Diagnostic tool	Number of studies	Pooled effect estimate and 95% confidence intervals	Heterogeneity (%)	Comparisons of MRI and PET or PET/CT
Sensitivity	Study design	Prospective	MRI	19	0.85 (0.76-0.91)	72.43	1.05 (0.94-1.17); P=0.373
			PET or PET/CT	27	0.81 (0.76-0.85)	47.17	
		Retrospective	MRI	22	0.81 (0.71-0.89)	78.85	1.01 (0.86-1.19); P=0.880
			PET or PET/CT	11	0.80 (0.70-0.88)	59.43	
	Sample size	>50	MRI	18	0.81 (0.67-0.90)	85.78	1.04 (0.88-1.22); P=0.652
			PET or PET/CT	19	0.78 (0.72-0.83)	41.13	
		<50	MRI	23	0.84 (0.78-0.89)	38.70	0.99 (0.89-1.09); P=0.820
			PET or PET/CT	19	0.85 (0.77-0.90)	56.94	
	Mean age (years)	>60	MRI	28	0.85 (0.78-0.89)	67.49	1.09 (0.99-1.19); P=0.067
			PET or PET/CT	25	0.78 (0.73-0.83)	45.42	
		<60	MRI	11	0.82 (0.67-0.91)	78.05	0.94 (0.79-1.13); P=0.519
			PET or PET/CT	11	0.87 (0.77-0.93)	54.16	
Specificity	Study design	Prospective	MRI	19	0.83 (0.78-0.88)	51.97	1.05 (0.95-1.16); P=0.323
			PET or PET/CT	27	0.79 (0.72-0.84)	81.87	
		Retrospective	MRI	22	0.86 (0.77-0.92)	94.35	1.28 (1.06-1.55); P=0.010
			PET or PET/CT	11	0.67 (0.55-0.77)	78.72	
	Sample size	>50	MRI	18	0.87 (0.78-0.93)	95.85	1.14 (1.00-1.31); P=0.046
			PET or PET/CT	19	0.76 (0.68-0.83)	87.26	
		<50	MRI	23	0.82 (0.76-0.86)	38.76	1.11 (0.97-1.26); P=0.126
			PET or PET/CT	19	0.74 (0.65-0.82)	63.29	
	Mean age (years)	>60	MRI	28	0.82 (0.76-0.87)	89.81	1.11 (1.00-1.23); P=0.059
			PET or PET/CT	25	0.74 (0.67-0.79)	81.12	
		<60	MRI	11	0.89 (0.77-0.95)	91.49	1.14 (1.00-1.31); P=0.055
			PET or PET/CT	11	0.78 (0.71-0.84)	41.18	
PLR	Study design	Prospective	MRI	19	5.12 (3.90-6.71)	7.25	1.35 (0.92-1.98); P=0.124
			PET or PET/CT	27	3.79 (2.89-4.96)	76.87	
		Retrospective	MRI	22	5.80 (3.62-9.30)	93.61	2.39 (1.33-4.27); P=0.003
			PET or PET/CT	11	2.43 (1.73-3.41)	72.45	
	Sample size	>50	MRI	18	6.46 (3.82-10.93)	95.37	1.98 (1.08-3.61); P=0.027
			PET or PET/CT	19	3.27 (2.43-4.41)	80.47	
		<50	MRI	23	4.61 (3.53-6.03)	34.62	1.40 (0.92-2.15); P=0.121
			PET or PET/CT	19	3.29 (2.36-4.58)	81.15	
	Mean age (years)	>60	MRI	28	4.82 (3.60-6.47)	90.87	1.62 (1.11-2.38); P=0.013
			PET or PET/CT	25	2.97 (2.32-3.80)	71.85	
		<60	MRI	11	7.60 (3.43-16.85)	76.24	1.89 (0.80-4.44); P=0.144
			PET or PET/CT	11	4.02 (2.95-5.49)	0.00	
NLR	Study design	Prospective	MRI	19	0.18 (0.12-0.29)	85.37	0.75 (0.45-1.24); P=0.264
			PET or PET/CT	27	0.24 (0.19-0.31)	50.09	
		Retrospective	MRI	22	0.22 (0.14-0.33)	87.13	0.76 (0.41-1.42); P=0.385
			PET or PET/CT	11	0.29 (0.19-0.47)	59.87	
	Sample size	>50	MRI	18	0.22 (0.13-0.38)	92.36	0.76 (0.42-1.36); P=0.356
			PET or PET/CT	19	0.29 (0.23-0.37)	46.48	
		<50	MRI	23	0.19 (0.14-0.27)	36.22	0.95 (0.54-1.66); P=0.857
			PET or PET/CT	19	0.20 (0.13-0.32)	55.68	
	Mean age (years)	>60	MRI	28	0.19 (0.13-0.26)	91.75	0.63 (0.42-0.96); P=0.033
			PET or PET/CT	25	0.30 (0.23-0.37)	52.39	
		<60	MRI	11	0.20 (0.10-0.39)	88.41	1.18 (0.47-2.92); P=0.726
			PET or PET/CT	11	0.17 (0.09-0.30)	35.85	

## Discussion

The present meta-analysis was based on published studies and investigated the predictive value of MRI and FDG-PET for the response to NACT of patients with locally advanced RC. This comprehensive, quantitative study included 74 studies with 4,105 patients with a wide range of patients’ characteristics. Our findings suggest that MRI and FDG-PET had a moderate predictive value for the response to NACT. Moreover, the predictive value of MRI might be superior to that of FDG-PET, in terms of specificity, PLR, and AUC. Finally, the predictive value of MRI and FDG-PET for predicting the response to NACT of patients with locally advanced RC could affect by sample size, and mean age.

A previous meta-analysis of 33 studies was conducted to compare the predictive value of MRI and FDG-PET for the pathological response to NACT in patients with RC (102). Its authors found that MRI was superior to FDG-PET in predicting the pathological response to NACT, whereas the specificity and positive predictive value of MRI was relatively lower, especially in patients with mucinous-type rectal adenocarcinomas. However, several studies were not included in this analysis, and indirect comparisons of MRI and FDG-PET were not performed. Moreover, an important meta-analysis evaluated the diagnostic performance of MRI, endorectal ultrasonography, and CT in predicting the response to preoperative therapy for patients with locally advanced RC based on 46 studies. They suggested MRI, endorectal ultrasonography, and CT could not used to predict complete response to NACT, and the positive predictive value for above imaging techniques was low for evaluated the tumor invasion in the circumferential resection margin. Furthermore, the diagnostic accuracy of MRI, endorectal ultrasonography, and CT for the prediction of metastatic lymph node disease was low. However, the study pooled only the diagnostic parameters for each diagnostic tool, and no comparisons of these imaging techniques were conducted. In addition, no stratification of analyses based on study or patients’ characteristics was conducted (103). Therefore, the current meta-analysis was performed to compare the diagnostic value of MRI and FDG-PET for the response to NACT in patients with locally advanced RC.

The summarized diagnostic parameters of MRI were higher than those of FDG-PET in terms of sensitivity, specificity, PLR, and AUC; the NLR of MRI was lower than that of FDG-PET. Moreover, the diagnostic values of MRI and FDG-PET were moderate for the response to NACT. Moreover, an indirect comparisons results indicated MRI were associated with higher specificity, PLR, and AUC than FDG-PET. Nevertheless, these results require further prospective research for a more comprehensive update of the diagnostic values of MRI and FDG-PET for the response to NACT. Several advantages of MRI should be mentioned: (1) The application of MRI in patients with locally RC without ionizing radiation prevents the stimulation of tumor progression by ionizing radiation; (2) The ancillary equipment of cyclotron is not installed nearby, which is associated with a lower cost than that of FDG-PET/CT application (104); and (3) The examination time in MRI was shorter than that in FDG-PET/CT (105). Therefore, MRI should be largely employed for preoperative evaluation in patients with RC.

To explore the sources of substantial heterogeneity, meta-regression and subgroup analyses were conducted based on the study design, sample size, and mean age of patients. The results of the meta-regression analyses indicated that the sample size and the mean age might have contributed to a significant heterogeneity in the sensitivity of MRI. Moreover, our subgroup analyses indicated that MRI was superior to FDG-PET when the pooled study was designed as retrospective, with a larger sample size, and a mean age of patients > 60.0 years. The potential reasons for this discrepancy could the evidence level, weighted based on the overall analyses and the tumor stages are affecting by mean age.

Several strengths of our study should be highlighted. First, the large sample size allowed us to quantitatively compare the predictive values of MRI and FDG-PET for the response to NACT in patients with locally advanced RC. Thus, our findings are potentially more robust than those of any other earlier individual study. Second, the consistency of the findings of this investigation and the lack of significant publication bias also support the robustness of our present findings. Third, indirect comparisons of the predictive value of MRI and FDG-PET were conducted to provide a better imaging tool for the response to NACT. Finally, the present study provides evidence for evaluation of the diagnostic values of MRI and FDG-PET in patients with specific characteristics.

The limitations of our study are as follows: (1) A study designed as retrospective was included in this analysis, which might have introduced uncontrolled bias; (2) Inconsistencies in the characteristics were present among the included studies, especially in terms of tumor properties, which were not reported in most of the included studies; (3) The heterogeneity across included studies were not fully explained by subgroup analyses; (4) Although the imaging examination were performed after NACT and before surgery, while the exact timing of the scanning might play an important role on the diagnostic ability of MRI and FDG-PET; (5) Although our results indicated no significant publication bias, this study was based on published articles, and publication bias was inevitable; and (6) Stratified analyses based on additional characteristics of patients were not conducted since this information was not available.

## Conclusion

The results of this study show that MRI and FDG-PET have a moderate diagnostic ability for the response of patients with locally advanced RC to NACT. The results of our indirect analyses suggested MRI was associated with elevated specificity, PLR, and AUC than FDG-PET. Subgroup analyses indicated that the predictive value of MRI was superior to that FDG-PET when the pooled study was designed as retrospective, with a large sample size, and a mean age of the patients > 60.0 years. These results add to the existing evidence but need further prospective research that would perform direct comparisons between the predictive values of MRI and FDG-PET for the response of patients with locally advanced RC to NACT.

## Author contributions

PG performed data acquisition, analysis, and interpretation, drafted the article, and obtaining the final approval. NL was responsible for data acquisition, analysis, and interpretation, drafting of the article, and obtaining the final approval. WL developed the conception and design of the study, conducted critical revision, and participated in obtaining the final approval. All authors contributed to the article and approved the submitted version.

## References

[1] Siegel RL Miller KD Jemal A . Cancer statistics, 2017. CA Cancer J Clin (2017) 67:7–30. doi: 10.3322/caac.21387 28055103

[2] Whiting P Rutjes AW Reitsma JB Bossuyt PM Kleijnen J . The development of QUADAS: a tool for the quality assessment of studies of diagnostic accuracy included in systematic reviews. BMC Med Res Methodol (2003) 3:25. doi: 10.1186/1471-2288-3-25 14606960 PMC305345

[3] McCourt M Armitage J Monson JR . Rectal cancer. Surgeon (2009) 7:162–9. doi: 10.1016/s1479-666x(09)80040-1 19580180

[4] Maringe C Walters S Rachet B Butler J Fields T Finan P . Stage at diagnosis and colorectal cancer survival in six high-income countries: a population-based study of patients diagnosed during 2000-2007. Acta Oncol (2013) 52:919–32. doi: 10.3109/0284186x.2013.764008 23581611

[5] Cercek A Goodman KA Hajj C Weisberger E Segal NH Reidy-Lagunes DL . Neoadjuvant chemotherapy first, followed by chemoradiation and then surgery, in the management of locally advanced rectal cancer. J Natl Compr Canc Netw (2014) 12:513–9. doi: 10.6004/jnccn.2014.0056 PMC5612781 24717570

[6] Fokas E Liersch T Fietkau R Hohenberger W Beissbarth T Hess C . Tumor regression grading after preoperative chemoradiotherapy for locally advanced rectal carcinoma revisited: updated results of the CAO/ARO/AIO-94 trial. J Clin Oncol (2014) 32:1554–62. doi: 10.1200/jco.2013.54.3769 24752056

[7] Sauer R Becker H Hohenberger W Rödel C Wittekind C Fietkau R . Preoperative versus postoperative chemoradiotherapy for rectal cancer. N Engl J Med (2004) 351:1731–40. doi: 10.1056/NEJMoa040694 15496622

[8] Bosset JF Collette L Calais G Mineur L Maingon P Radosevic-Jelic L . Chemotherapy with preoperative radiotherapy in rectal cancer. N Engl J Med (2006) 355:1114–23. doi: 10.1056/NEJMoa060829 16971718

[9] Habr-Gama A Perez RO Nadalin W Sabbaga J Ribeiro U Jr. Silva e Sousa AH Jr. . Operative versus nonoperative treatment for stage 0 distal rectal cancer following chemoradiation therapy: long-term results. Ann Surg (2004) 240:711–7. doi: 10.1097/01.sla.0000141194.27992.32 PMC1356472 15383798

[10] Habr-Gama A Perez RO Proscurshim I Campos FG Nadalin W Kiss D . Patterns of failure and survival for nonoperative treatment of stage c0 distal rectal cancer following neoadjuvant chemoradiation therapy. J Gastrointest Surg (2006) 10:1319–28 discussion 28-9. doi: 10.1016/j.gassur.2006.09.005 17175450

[11] Maas M Beets-Tan RG Lambregts DM Lammering G Nelemans PJ Engelen SM . Wait-and-see policy for clinical complete responders after chemoradiation for rectal cancer. J Clin Oncol (2011) 29:4633–40. doi: 10.1200/jco.2011.37.7176 22067400

[12] Appelt AL Pløen J Harling H Jensen FS Jensen LH Jørgensen JC . High-dose chemoradiotherapy and watchful waiting for distal rectal cancer: a prospective observational study. Lancet Oncol (2015) 16:919–27. doi: 10.1016/s1470-2045(15)00120-5 26156652

[13] Gollub MJ Tong T Weiser M Zheng J Gonen M Zakian KL . Limited accuracy of DCE-MRI in identification of pathological complete responders after chemoradiotherapy treatment for rectal cancer. Eur Radiol (2017) 27:1605–12. doi: 10.1007/s00330-016-4493-1 PMC5570543 27436029

[14] Zhang C Tong J Sun X Liu J Wang Y Huang G . 18F-FDG-PET evaluation of treatment response to neo-adjuvant therapy in patients with locally advanced rectal cancer: a meta-analysis. Int J Cancer (2012) 131:2604–11. doi: 10.1002/ijc.27557 22447461

[15] Dzik-Jurasz A Domenig C George M Wolber J Padhani A Brown G . Diffusion MRI for prediction of response of rectal cancer to chemoradiation. Lancet (2002) 360:307–8. doi: 10.1016/s0140-6736(02)09520-x 12147376

[16] Joyce DL Wahl RL Patel PV Schulick RD Gearhart SL Choti MA . Preoperative positron emission tomography to evaluate potentially resectable hepatic colorectal metastases. Arch Surg (2006) 141:1220–6 discussion 27. doi: 10.1001/archsurg.141.12.1220 17178965

[17] Ogunbiyi OA Flanagan FL Dehdashti F Siegel BA Trask DD Birnbaum EH . Detection of recurrent and metastatic colorectal cancer: comparison of positron emission tomography and computed tomography. Ann Surg Oncol (1997) 4:613–20. doi: 10.1007/bf02303744 9416407

[18] Staib L Schirrmeister H Reske SN Beger HG . Is (18)F-fluorodeoxyglucose positron emission tomography in recurrent colorectal cancer a contribution to surgical decision making? Am J Surg (2000) 180:1–5. doi: 10.1016/s0002-9610(00)00406-2 11036130

[19] Even-Sapir E Parag Y Lerman H Gutman M Levine C Rabau M . Detection of recurrence in patients with rectal cancer: PET/CT after abdominoperineal or anterior resection. Radiology (2004) 232:815–22. doi: 10.1148/radiol.2323031065 15273334

[20] Cohade C Osman M Leal J Wahl RL . Direct comparison of (18)F-FDG PET and PET/CT in patients with colorectal carcinoma. J Nucl Med (2003) 44:1797–803. 14602862

[21] Moher D Liberati A Tetzlaff J Altman DG . Preferred reporting items for systematic reviews and meta-analyses: the PRISMA statement. PLoS Med (2009) 6:e1000097. doi: 10.1371/journal.pmed.1000097 19621072 PMC2707599

[22] DerSimonian R Laird N . Meta-analysis in clinical trials. Control Clin Trials (1986) 7:177–88. doi: 10.1016/0197-2456(86)90046-2 3802833

[23] Walter SD . Properties of the summary receiver operating characteristic (SROC) curve for diagnostic test data. Stat Med (2002) 21:1237–56. doi: 10.1002/sim.1099 12111876

[24] Chu H Cole SR . Bivariate meta-analysis of sensitivity and specificity with sparse data: a generalized linear mixed model approach. J Clin Epidemiol (2006) 59:1331–2. doi: 10.1016/j.jclinepi.2006.06.011. author reply 1332-3. 17098577

[25] Higgins JP Thompson SG Deeks JJ Altman DG . Measuring inconsistency in meta-analyses. BMJ (2003) 327:557–60. doi: 10.1136/bmj.327.7414.557 PMC192859 12958120

[26] Woodward M . Epidemiology: Study design and data analysis. 2nd edn. Boca Raton, FL, USA: Chapman and Hall/CRC (2005).

[27] Deeks JJ Macaskill P Irwig L . The performance of tests of publication bias and other sample size effects in systematic reviews of diagnostic test accuracy was assessed. J Clin Epidemiol (2005) 58:882–93. doi: 10.1016/j.jclinepi.2005.01.016 16085191

[28] Amthauer H Denecke T Rau B Hildebrandt B Hünerbein M Ruf J . Response prediction by FDG-PET after neoadjuvant radiochemotherapy and combined regional hyperthermia of rectal cancer: correlation with endorectal ultrasound and histopathology. Eur J Nucl Med Mol Imaging (2004) 31:811–9. doi: 10.1007/s00259-003-1453-1 14762698

[29] Capirci C Rubello D Chierichetti F Crepaldi G Carpi A Nicolini A . Restaging after neoadjuvant chemoradiotherapy for rectal adenocarcinoma: Role of F18-FDG PET. BioMed Pharmacother (2004) 58:451–7. doi: 10.1016/j.biopha.2004.08.005 15464875

[30] Denecke T Rau B Hoffmann KT Hildebrandt B Ruf J Gutberlet M . Comparison of CT, MRI and FDG-PET in response prediction of patients with locally advanced rectal cancer after multimodal preoperative therapy: is there a benefit in using functional imaging? Eur Radiol (2005) 15:1658–66. doi: 10.1007/s00330-005-2658-4 15806369

[31] Cascini GL Avallone A Delrio P Guida C Tatangelo F Marone P . 18F-FDG PET is an early predictor of pathologic tumor response to preoperative radiochemotherapy in locally advanced rectal cancer. J Nucl Med (2006) 47:1241–8. 16883000

[32] Melton GB Lavely WC Jacene HA Schulick RD Choti MA Wahl RL . Efficacy of preoperative combined 18-fluorodeoxyglucose positron emission tomography and computed tomography for assessing primary rectal cancer response to neoadjuvant therapy. J Gastrointest Surg (2007) 11:961–9 discussion 69. doi: 10.1007/s11605-007-0170-7 17541799

[33] Kristiansen C Loft A Berthelsen AK Graff J Lindebjerg J Bisgaard C . PET/CT and histopathologic response to preoperative chemoradiation therapy in locally advanced rectal cancer. Dis Colon Rectum (2008) 51:21–5. doi: 10.1007/s10350-007-9095-1 17975715

[34] Capirci C Rubello D Pasini F Galeotti F Bianchini E Del Favero G . The role of dual-time combined 18-fluorodeoxyglucose positron emission tomography and computed tomography in the staging and restaging workup of locally advanced rectal cancer, treated with preoperative chemoradiation therapy and radical surgery. Int J Radiat Oncol Biol Phys (2009) 74:1461–9. doi: 10.1016/j.ijrobp.2008.10.064 19419820

[35] Rosenberg R Herrmann K Gertler R Künzli B Essler M Lordick F . The predictive value of metabolic response to preoperative radiochemotherapy in locally advanced rectal cancer measured by PET/CT. Int J Colorectal Dis (2009) 24:191–200. doi: 10.1007/s00384-008-0616-8 19050900

[36] Palma P Conde-Muíño R Rodríguez-Fernández A Segura-Jiménez I Sánchez-Sánchez R Martín-Cano J . The value of metabolic imaging to predict tumour response after chemoradiation in locally advanced rectal cancer. Radiat Oncol (2010) 5:119. doi: 10.1186/1748-717x-5-119 21159200 PMC3012041

[37] Lambrecht M Deroose C Roels S Vandecaveye V Penninckx F Sagaert X . The use of FDG-PET/CT and diffusion-weighted magnetic resonance imaging for response prediction before, during and after preoperative chemoradiotherapy for rectal cancer. Acta Oncol (2010) 49:956–63. doi: 10.3109/0284186x.2010.498439 20586658

[38] Martoni AA Di Fabio F Pinto C Castellucci P Pini S Ceccarelli C . Prospective study on the FDG-PET/CT predictive and prognostic values in patients treated with neoadjuvant chemoradiation therapy and radical surgery for locally advanced rectal cancer. Ann Oncol (2011) 22:650–56. doi: 10.1093/annonc/mdq433 20847032

[39] Hur H Kim NK Yun M Min BS Lee KY Keum KC . 18Fluoro-deoxy-glucose positron emission tomography in assessing tumor response to preoperative chemoradiation therapy for locally advanced rectal cancer. J Surg Oncol (2011) 103:17–24. doi: 10.1002/jso.21736 20886551

[40] Yoon MS Ahn SJ Nah BS Chung WK Song JY Jeong JU . The metabolic response using 18F-fluorodeoxyglucose-positron emission tomography/computed tomography and the change in the carcinoembryonic antigen level for predicting response to pre-operative chemoradiotherapy in patients with rectal cancer. Radiother Oncol (2011) 98:134–8. doi: 10.1016/j.radonc.2010.10.012 21040991

[41] Kim YC Lim JS Keum KC Kim KA Myoung S Shin SJ . Comparison of diffusion-weighted MRI and MR volumetry in the evaluation of early treatment outcomes after preoperative chemoradiotherapy for locally advanced rectal cancer. J Magn Reson Imaging (2011) 34:570–6. doi: 10.1002/jmri.22696 21751285

[42] Kim SH Lee JY Lee JM Han JK Choi BI . Apparent diffusion coefficient for evaluating tumour response to neoadjuvant chemoradiation therapy for locally advanced rectal cancer. Eur Radiol (2011) 21:987–95. doi: 10.1007/s00330-010-1989-y 20978768

[43] Herrmann K Bundschuh RA Rosenberg R Schmidt S Praus C Souvatzoglou M . Comparison of different SUV-based methods for response prediction to neoadjuvant radiochemotherapy in locally advanced rectal cancer by FDG-PET and MRI. Mol Imaging Biol (2011) 13:1011–9. doi: 10.1007/s11307-010-0383-0 20936364

[44] Guerra L Niespolo R Di Pisa G Ippolito D De Ponti E Terrevazzi S . Change in glucose metabolism measured by 18F-FDG PET/CT as a predictor of histopathologic response to neoadjuvant treatment in rectal cancer. Abdom Imaging (2011) 36:38–45. doi: 10.1007/s00261-009-9594-8 20033405

[45] Everaert H Hoorens A Vanhove C Sermeus A Ceulemans G Engels B . Prediction of response to neoadjuvant radiotherapy in patients with locally advanced rectal cancer by means of sequential 18FDG-PET. Int J Radiat Oncol Biol Phys (2011) 80:91–6. doi: 10.1016/j.ijrobp.2010.01.021 20605358

[46] Curvo-Semedo L Lambregts DM Maas M Thywissen T Mehsen RT Lammering G . Rectal cancer: assessment of complete response to preoperative combined radiation therapy with chemotherapy–conventional MR volumetry versus diffusion-weighted MR imaging. Radiology (2011) 260:734–43. doi: 10.1148/radiol.11102467 21673229

[47] Song I Kim SH Lee SJ Choi JY Kim MJ Rhim H . Value of diffusion-weighted imaging in the detection of viable tumour after neoadjuvant chemoradiation therapy in patients with locally advanced rectal cancer: comparison with T2 weighted and PET/CT imaging. Br J Radiol (2012) 85:577–86. doi: 10.1259/bjr/68424021 PMC3479876 21343320

[48] Ippolito D Monguzzi L Guerra L Deponti E Gardani G Messa C . Response to neoadjuvant therapy in locally advanced rectal cancer: assessment with diffusion-weighted MR imaging and 18FDG PET/CT. Abdom Imaging (2012) 37:1032–40. doi: 10.1007/s00261-011-9839-1 22270580

[49] Perez RO Habr-Gama A Gama-Rodrigues J Proscurshim I Julião GP Lynn P . Accuracy of positron emission tomography/computed tomography and clinical assessment in the detection of complete rectal tumor regression after neoadjuvant chemoradiation: long-term results of a prospective trial (National clinical trial 00254683). Cancer (2012) 118:3501–11. doi: 10.1002/cncr.26644 22086847

[50] Lambrecht M Vandecaveye V De Keyzer F Roels S Penninckx F Van Cutsem E . Value of diffusion-weighted magnetic resonance imaging for prediction and early assessment of response to neoadjuvant radiochemotherapy in rectal cancer: preliminary results. Int J Radiat Oncol Biol Phys (2012) 82:863–70. doi: 10.1016/j.ijrobp.2010.12.063 21398048

[51] Jung SH Heo SH Kim JW Jeong YY Shin SS Soung MG . Predicting response to neoadjuvant chemoradiation therapy in locally advanced rectal cancer: Diffusion-weighted 3 Tesla MR imaging. J Magn Reson Imaging (2012) 35:110–6. doi: 10.1002/jmri.22749 21989997

[52] Janssen MH Öllers MC van Stiphout RG Riedl RG van den Bogaard J Buijsen J . PET-based treatment response evaluation in rectal cancer: prediction and validation. Int J Radiat Oncol Biol Phys (2012) 82:871–6. doi: 10.1016/j.ijrobp.2010.11.038 21377810

[53] Huh JW Min JJ Lee JH Kim HR Kim YJ . The predictive role of sequential FDG-PET/CT in response of locally advanced rectal cancer to neoadjuvant chemoradiation. Am J Clin Oncol (2012) 35:340–4. doi: 10.1097/COC.0b013e3182118e7d 21422901

[54] Chennupati SK Quon A Kamaya A Pai RK La T Krakow TE . Positron emission tomography for predicting pathologic response after neoadjuvant chemoradiotherapy for locally advanced rectal cancer. Am J Clin Oncol (2012) 35:334–9. doi: 10.1097/COC.0b013e3182118d12 21422989

[55] Barbaro B Vitale R Valentini V Illuminati S Vecchio FM Rizzo G . Diffusion-weighted magnetic resonance imaging in monitoring rectal cancer response to neoadjuvant chemoradiotherapy. Int J Radiat Oncol Biol Phys (2012) 83:594–9. doi: 10.1016/j.ijrobp.2011.07.017 22099033

[56] Guillem JG Ruby JA Leibold T Akhurst TJ Yeung HW Gollub MJ . Neither FDG-PET nor CT can distinguish between a pathological complete response and an incomplete response after neoadjuvant chemoradiation in locally advanced rectal cancer: a prospective study. Ann Surg (2013) 258:289–95. doi: 10.1097/SLA.0b013e318277b625 PMC12432588 23187748

[57] Hatt M van Stiphout R le Pogam A Lammering G Visvikis D Lambin P . Early prediction of pathological response in locally advanced rectal cancer based on sequential 18F-FDG PET. Acta Oncol (2013) 52:619–26. doi: 10.3109/0284186x.2012.702923 PMC4873546 22873767

[58] Murcia Duréndez MJ Frutos Esteban L Luján J Frutos MD Valero G Navarro Fernández JL . The value of 18F-FDG PET/CT for assessing the response to neoadjuvant therapy in locally advanced rectal cancer. Eur J Nucl Med Mol Imaging (2013) 40:91–7. doi: 10.1007/s00259-012-2257-y 23081822

[59] Calvo FA Sole CV de la Mata D Cabezón L Gómez-Espí M Alvarez E . ^18^F-FDG PET/CT-based treatment response evaluation in locally advanced rectal cancer: a prospective validation of long-term outcomes. Eur J Nucl Med Mol Imaging (2013) 40:657–67. doi: 10.1007/s00259-013-2341-y 23436067

[60] Sun W Xu J Hu W Zhang Z Shen W . The role of sequential 18(F) -FDG PET/CT in predicting tumour response after preoperative chemoradiation for rectal cancer. Colorectal Dis (2013) 15:e231–8. doi: 10.1111/codi.12165 23384167

[61] Genovesi D Filippone A Ausili Cèfaro G Trignani M Vinciguerra A Augurio A . Diffusion-weighted magnetic resonance for prediction of response after neoadjuvant chemoradiation therapy for locally advanced rectal cancer: preliminary results of a monoinstitutional prospective study. Eur J Surg Oncol (2013) 39:1071–8. doi: 10.1016/j.ejso.2013.07.090 23953231

[62] Park J Chang KJ Seo YS Byun BH Choi JH Moon H . Tumor SUVmax normalized to liver uptake on (18)F-FDG PET/CT predicts the pathologic complete response after neoadjuvant chemoradiotherapy in locally advanced rectal cancer. Nucl Med Mol Imaging (2014) 48:295–302. doi: 10.1007/s13139-014-0289-x 26396634 PMC4571668

[63] Niccoli-Asabella A Altini C De Luca R Fanelli M Rubini D Caliandro C . Prospective analysis of 18F-FDG PET/CT predictive value in patients with low rectal cancer treated with neoadjuvant chemoradiotherapy and conservative surgery. BioMed Res Int (2014) 2014:952843. doi: 10.1155/2014/952843 24877151 PMC4024401

[64] Cai PQ Wu YP An X Qiu X Kong LH Liu GC . Simple measurements on diffusion-weighted MR imaging for assessment of complete response to neoadjuvant chemoradiotherapy in locally advanced rectal cancer. Eur Radiol (2014) 24:2962–70. doi: 10.1007/s00330-014-3251-5 25038851

[65] Aiba T Uehara K Nihashi T Tsuzuki T Yatsuya H Yoshioka Y . MRI And FDG-PET for assessment of response to neoadjuvant chemotherapy in locally advanced rectal cancer. Ann Surg Oncol (2014) 21:1801–8. doi: 10.1245/s10434-014-3538-4 24531702

[66] Doi H Beppu N Kato T Noda M Yanagi H Tomita N . Diffusion-weighted magnetic resonance imaging for prediction of tumor response to neoadjuvant chemoradiotherapy using irinotecan plus s-1 for rectal cancer. Mol Clin Oncol (2015) 3:1129–34. doi: 10.3892/mco.2015.604 PMC4535051 26623064

[67] Blažić I Maksimović R Gajić M Šaranović Đ . Apparent diffusion coefficient measurement covering complete tumor area better predicts rectal cancer response to neoadjuvant chemoradiotherapy. Croat Med J (2015) 56:460–9. doi: 10.3325/cmj.2015.56.460 PMC4655931 26526883

[68] Martens MH van Heeswijk MM van den Broek JJ Rao SX Vandecaveye V Vliegen RA . Prospective, multicenter validation study of magnetic resonance volumetry for response assessment after preoperative chemoradiation in rectal cancer: Can the results in the literature be reproduced? Int J Radiat Oncol Biol Phys (2015) 93:1005–14. doi: 10.1016/j.ijrobp.2015.09.008 26581139

[69] Petrillo M Fusco R Catalano O Sansone M Avallone A Delrio P . MRI For assessing response to neoadjuvant therapy in locally advanced rectal cancer using DCE-MR and DW-MR data sets: A preliminary report. BioMed Res Int (2015) 2015:514740. doi: 10.1155/2015/514740 26413528 PMC4564611

[70] Choi MH Oh SN Rha SE Choi JI Lee SH Jang HS . Diffusion-weighted imaging: Apparent diffusion coefficient histogram analysis for detecting pathologic complete response to chemoradiotherapy in locally advanced rectal cancer. J Magn Reson Imaging (2016) 44:212–20. doi: 10.1002/jmri.25117 26666560

[71] Leccisotti L Gambacorta MA de Waure C Stefanelli A Barbaro B Vecchio FM . The predictive value of 18F-FDG PET/CT for assessing pathological response and survival in locally advanced rectal cancer after neoadjuvant radiochemotherapy. Eur J Nucl Med Mol Imaging (2015) 42:657–66. doi: 10.1007/s00259-014-2820-9 25687534

[72] Tong T Sun Y Gollub MJ Peng W Cai S Zhang Z . Dynamic contrast-enhanced MRI: Use in predicting pathological complete response to neoadjuvant chemoradiation in locally advanced rectal cancer. J Magn Reson Imaging (2015) 42:673–80. doi: 10.1002/jmri.24835 25652254

[73] Martens MH Subhani S Heijnen LA Lambregts DM Buijsen J Maas M . Can perfusion MRI predict response to preoperative treatment in rectal cancer? Radiother Oncol (2015) 114:218–23. doi: 10.1016/j.radonc.2014.11.044 25497874

[74] Altini C Niccoli Asabella A De Luca R Fanelli M Caliandro C Quartuccio N . Comparison of (18)F-FDG PET/CT methods of analysis for predicting response to neoadjuvant chemoradiation therapy in patients with locally advanced low rectal cancer. Abdom Imaging (2015) 40:1190–202. doi: 10.1007/s00261-014-0277-8 25348731

[75] Lambregts DM Rao SX Sassen S Martens MH Heijnen LA Buijsen J . MRI And diffusion-weighted MRI volumetry for identification of complete tumor responders after preoperative chemoradiotherapy in patients with rectal cancer: A bi-institutional validation study. Ann Surg (2015) 262:1034–9. doi: 10.1097/sla.0000000000000909 25211270

[76] Koo PJ Kim SJ Chang S Kwak JJ . Interim fluorine-18 fluorodeoxyglucose positron emission Tomography/Computed tomography to predict pathologic response to preoperative chemoradiotherapy and prognosis in patients with locally advanced rectal cancer. Clin Colorectal Cancer (2016) 15:e213–e19. doi: 10.1016/j.clcc.2016.04.002 27316919

[77] Travaini LL Zampino MG Colandrea M Ferrari ME Gilardi L Leonardi MC . PET/CT with fluorodeoxyglucose during neoadjuvant chemoradiotherapy in locally advanced rectal cancer. Ecancermedicalscience (2016) 10:629. doi: 10.3332/ecancer.2016.629 27110285 PMC4817524

[78] Li QW Zheng RL Ling YH Wang QX Xiao WW Zeng ZF . Prediction of tumor response after neoadjuvant chemoradiotherapy in rectal cancer using (18)fluorine-2-deoxy-D-glucose positron emission tomography-computed tomography and serum carcinoembryonic antigen: a prospective study. Abdom Radiol (NY) (2016) 41:1448–55. doi: 10.1007/s00261-016-0698-7 27116012

[79] De Cecco CN Ciolina M Caruso D Rengo M Ganeshan B Meinel FG . Performance of diffusion-weighted imaging, perfusion imaging, and texture analysis in predicting tumoral response to neoadjuvant chemoradiotherapy in rectal cancer patients studied with 3T MR: initial experience. Abdom Radiol (NY) (2016) 41:1728–35. doi: 10.1007/s00261-016-0733-8 27056748

[80] Chen YG Chen MQ Guo YY Li SC Wu JX Xu BH . Apparent diffusion coefficient predicts pathology complete response of rectal cancer treated with neoadjuvant chemoradiotherapy. PloS One (2016) 11:e0153944. doi: 10.1371/journal.pone.0153944 27100991 PMC4839695

[81] Iannicelli E Di Pietropaolo M Pilozzi E Osti MF Valentino M Masoni L . Value of diffusion-weighted MRI and apparent diffusion coefficient measurements for predicting the response of locally advanced rectal cancer to neoadjuvant chemoradiotherapy. Abdom Radiol (NY) (2016) 41:1906–17. doi: 10.1007/s00261-016-0805-9 27323759

[82] Sathyakumar K Chandramohan A Masih D Jesudasan MR Pulimood A Eapen A . Best MRI predictors of complete response to neoadjuvant chemoradiation in locally advanced rectal cancer. Br J Radiol (2016) 89:20150328. doi: 10.1259/bjr.20150328 26828967 PMC4846192

[83] Jacobs L Intven M van Lelyveld N Philippens M Burbach M Seldenrijk K . Diffusion-weighted MRI for early prediction of treatment response on preoperative chemoradiotherapy for patients with locally advanced rectal cancer: A feasibility study. Ann Surg (2016) 263:522–8. doi: 10.1097/sla.0000000000001311 26106836

[84] Petrillo A Fusco R Granata V Setola SV Sansone M Rega D . MR imaging perfusion and diffusion analysis to assess preoperative short course radiotherapy response in locally advanced rectal cancer: Standardized index of shape by DCE-MRI and intravoxel incoherent motion-derived parameters by DW-MRI. Med Oncol (2017) 34:198. doi: 10.1007/s12032-017-1059-2 29151142

[85] Bassaneze T Gonçalves JE Faria JF Palma RT Waisberg J . Quantitative aspects of diffusion-weighted magnetic resonance imaging in rectal cancer response to neoadjuvant therapy. Radiol Oncol (2017) 51:270–76. doi: 10.1515/raon-2017-0025 PMC5611991 28959163

[86] De Felice F Magnante AL Musio D Ciolina M De Cecco CN Rengo M . Diffusion-weighted magnetic resonance imaging in locally advanced rectal cancer treated with neoadjuvant chemoradiotherapy. Eur J Surg Oncol (2017) 43:1324–29. doi: 10.1016/j.ejso.2017.03.010 28363512

[87] Zhu HB Zhang XY Zhou XH Li XT Liu YL Wang S . Assessment of pathological complete response to preoperative chemoradiotherapy by means of multiple mathematical models of diffusion-weighted MRI in locally advanced rectal cancer: A prospective single-center study. J Magn Reson Imaging (2017) 46:175–83. doi: 10.1002/jmri.25567 27981667

[88] Yu J Xu Q Song JC Li Y Dai X Huang DY . The value of diffusion kurtosis magnetic resonance imaging for assessing treatment response of neoadjuvant chemoradiotherapy in locally advanced rectal cancer. Eur Radiol (2017) 27:1848–57. doi: 10.1007/s00330-016-4529-6 27631106

[89] Petrillo A Fusco R Granata V Filice S Sansone M Rega D . Assessing response to neo-adjuvant therapy in locally advanced rectal cancer using intra-voxel incoherent motion modelling by DWI data and standardized index of shape from DCE-MRI. Ther Adv Med Oncol (2018) 10:1758835918809875. doi: 10.1177/1758835918809875 30479672 PMC6243411

[90] Fusco R Sansone M Granata V Grimm R Pace U Delrio P . Diffusion and perfusion MR parameters to assess preoperative short-course radiotherapy response in locally advanced rectal cancer: a comparative explorative study among standardized index of shape by DCE-MRI, intravoxel incoherent motion- and diffusion kurtosis imaging-derived parameters. Abdom Radiol (NY) (2019) 44:3683–700. doi: 10.1007/s00261-018-1801-z 30361867

[91] Murata H Okamoto M Takahashi T Motegi M Ogoshi K Shoji H . SUV(max)-based parameters of FDG-PET/CT reliably predict pathologic complete response after preoperative hyperthermo-chemoradiotherapy in rectal cancer. Anticancer Res (2018) 38:5909–16. doi: 10.21873/anticanres.12935 30275218

[92] Liu S Zhong GX Zhou WX Xue HD Pan WD Xu L . Can endorectal ultrasound, MRI, and mucosa integrity accurately predict the complete response for mid-low rectal cancer after preoperative chemoradiation? a prospective observational study from a single medical center. Dis Colon Rectum (2018) 61:903–10. doi: 10.1097/dcr.0000000000001135 29944579

[93] Aker M Boone D Chandramohan A Sizer B Motson R Arulampalam T . Diagnostic accuracy of MRI in assessing tumor regression and identifying complete response in patients with locally advanced rectal cancer after neoadjuvant treatment. Abdom Radiol (NY) (2018) 43:3213–19. doi: 10.1007/s00261-018-1627-8 29767284

[94] Horvat N Veeraraghavan H Khan M Blazic I Zheng J Capanu M . MR imaging of rectal cancer: Radiomics analysis to assess treatment response after neoadjuvant therapy. Radiology (2018) 287:833–43. doi: 10.1148/radiol.2018172300 PMC5978457 29514017

[95] Delli Pizzi A Cianci R Genovesi D Esposito G Timpani M Tavoletta A . Performance of diffusion-weighted magnetic resonance imaging at 3.0T for early assessment of tumor response in locally advanced rectal cancer treated with preoperative chemoradiation therapy. Abdom Radiol (NY) (2018) 43:2221–30. doi: 10.1007/s00261-018-1457-8 29332248

[96] Nahas SC Nahas CSR Cama GM de Azambuja RL Horvat N Marques CFS . Diagnostic performance of magnetic resonance to assess treatment response after neoadjuvant therapy in patients with locally advanced rectal cancer. Abdom Radiol (NY) (2019) 44:3632–40. doi: 10.1007/s00261-019-01894-8 30663025

[97] Giannini V Mazzetti S Bertotto I Chiarenza C Cauda S Delmastro E . Predicting locally advanced rectal cancer response to neoadjuvant therapy with (18)F-FDG PET and MRI radiomics features. Eur J Nucl Med Mol Imaging (2019) 46:878–88. doi: 10.1007/s00259-018-4250-6 30637502

[98] Palmisano A Di Chiara A Esposito A Rancoita PMV Fiorino C Passoni P . MRI Prediction of pathological response in locally advanced rectal cancer: when apparent diffusion coefficient radiomics meets conventional volumetry. Clin Radiol (2020) 75:798.e1–798.e11. doi: 10.1016/j.crad.2020.06.023 32712007

[99] Bae H Seo N Han K Koom WS Kim MJ Kim NK . MR prediction of pathologic complete response and early-stage rectal cancer after neoadjuvant chemoradiation in patients with clinical T1/T2 rectal cancer for organ saving strategy. Med (Baltimore) (2020) 99:e22746. doi: 10.1097/MD.0000000000022746 PMC7571887 33080736

[100] López-López V Abrisqueta Carrión J Luján J B Lynn P Frutos L Ono A . Assessing tumor response to neoadjuvant chemoradiation in rectal cancer with rectoscopy and 18F-FDG PET/CT: results from a prospective series. Rev Esp Enferm Dig (2021) 113:307–12. doi: 10.17235/reed.2020.6954/2020 33054291

[101] Uemura M Ikeda M Handa R Danno K Nishimura J Hata T . The efficiency of 18F-FDG-PET/CT in the assessment of tumor response to preoperative chemoradiation therapy for locally recurrent rectal cancer. BMC Cancer (2021) 21:1132. doi: 10.1186/s12885-021-08873-7 34674666 PMC8529852

[102] Li YL Wu LM Chen XX Delproposto Z Hu JN Xu JR . Is diffusion-weighted MRI superior to FDG-PET or FDG-PET/CT in evaluating and predicting pathological response to preoperative neoadjuvant therapy in patients with rectal cancer? J Dig Dis (2014) 15:525–37. doi: 10.1111/1751-2980.12174 25060294

[103] de Jong EA ten Berge JC Dwarkasing RS Rijkers AP van Eijck CH . The accuracy of MRI, endorectal ultrasonography, and computed tomography in predicting the response of locally advanced rectal cancer after preoperative therapy: A metaanalysis. Surgery (2016) 159:688–99. doi: 10.1016/j.surg.2015.10.019 26619929

[104] Usuda K Zhao XT Sagawa M Matoba M Kuginuki Y Taniguchi M . Diffusion-weighted imaging is superior to positron emission tomography in the detection and nodal assessment of lung cancers. Ann Thorac Surg (2011) 91:1689–95. doi: 10.1016/j.athoracsur.2011.02.037 21619964

[105] Nomori H Mori T Ikeda K Kawanaka K Shiraishi S Katahira K . Diffusion-weighted magnetic resonance imaging can be used in place of positron emission tomography for n staging of non-small cell lung cancer with fewer false-positive results. J Thorac Cardiovasc Surg (2008) 135:816–22. doi: 10.1016/j.jtcvs.2007.10.035 18374761

