# Supplementary material for: MRI VS. FDG-PET for diagnosis of response to neoadjuvant therapy in patients with locally advanced rectal cancer
Source: Front Oncol. 2023 Jan 18;13:1031581. doi: 10.3389/fonc.2023.1031581 (PMC9890074; doi:10.3389/fonc.2023.1031581)
Supplement: Supplementary file 1 [file DataSheet_1.docx]

**Supplemental 1**

**Search strategy in PubMed:**

("Magnetic Resonance Imaging"[Mesh] OR "Magnetic Resonance Imaging"[Title/Abstract]) OR ("Positron-Emission Tomography"[Mesh] OR "Positron-Emission Tomography"[Title/Abstract]) OR ("computed tomography"[Mesh] OR "computed tomography"[Title/Abstract]) AND (“rectal cancer”[Mesh] OR “rectal cancer”[Title/Abstract]) AND (“preoperative”[Mesh] OR “preoperative”[Title/Abstract] OR “neoadjuvant”[Mesh] OR “neoadjuvant”[Title/Abstract])

**Supplemental 2**


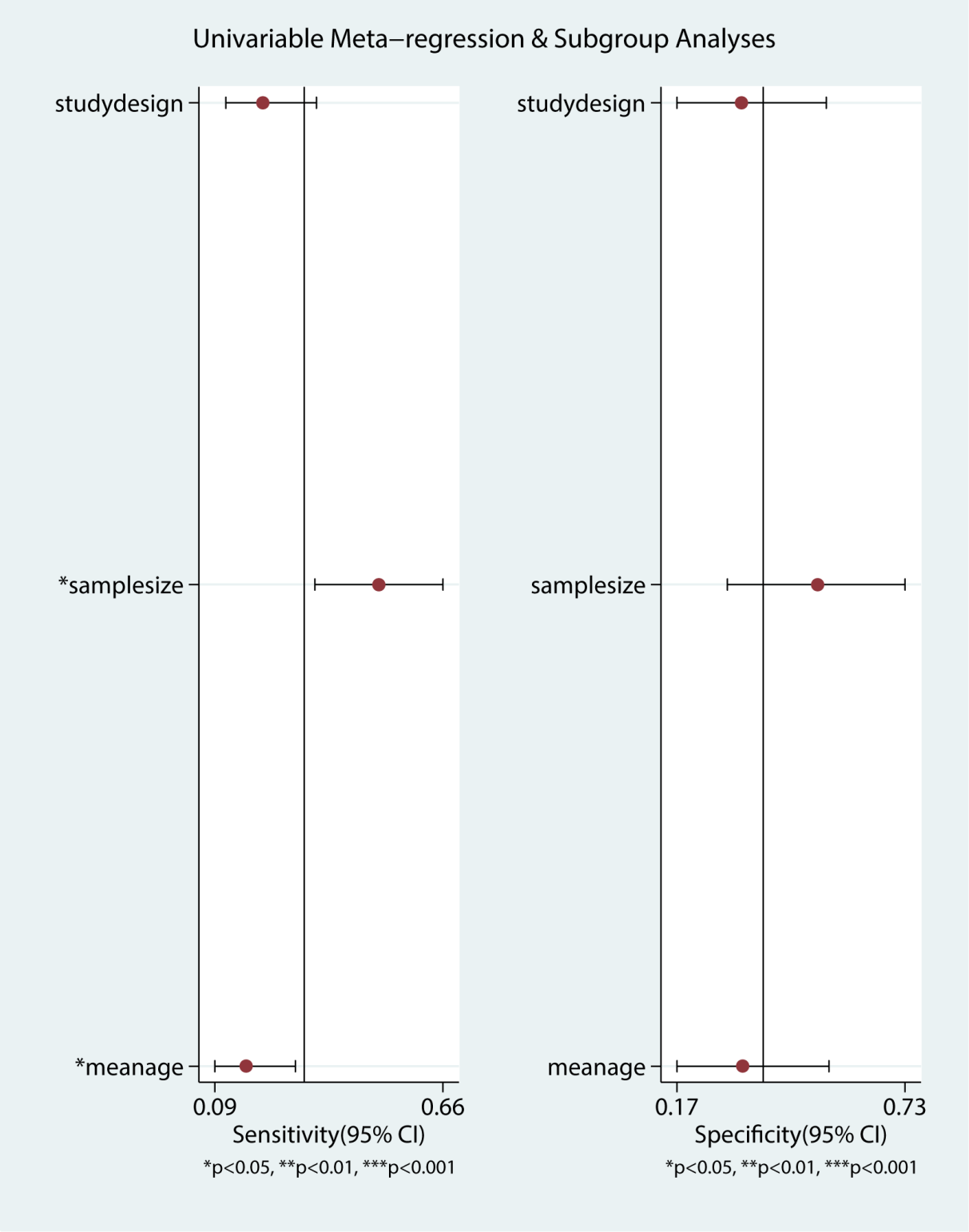


Figure S1. Meta-regression for MRI based on sensitivity and specificity


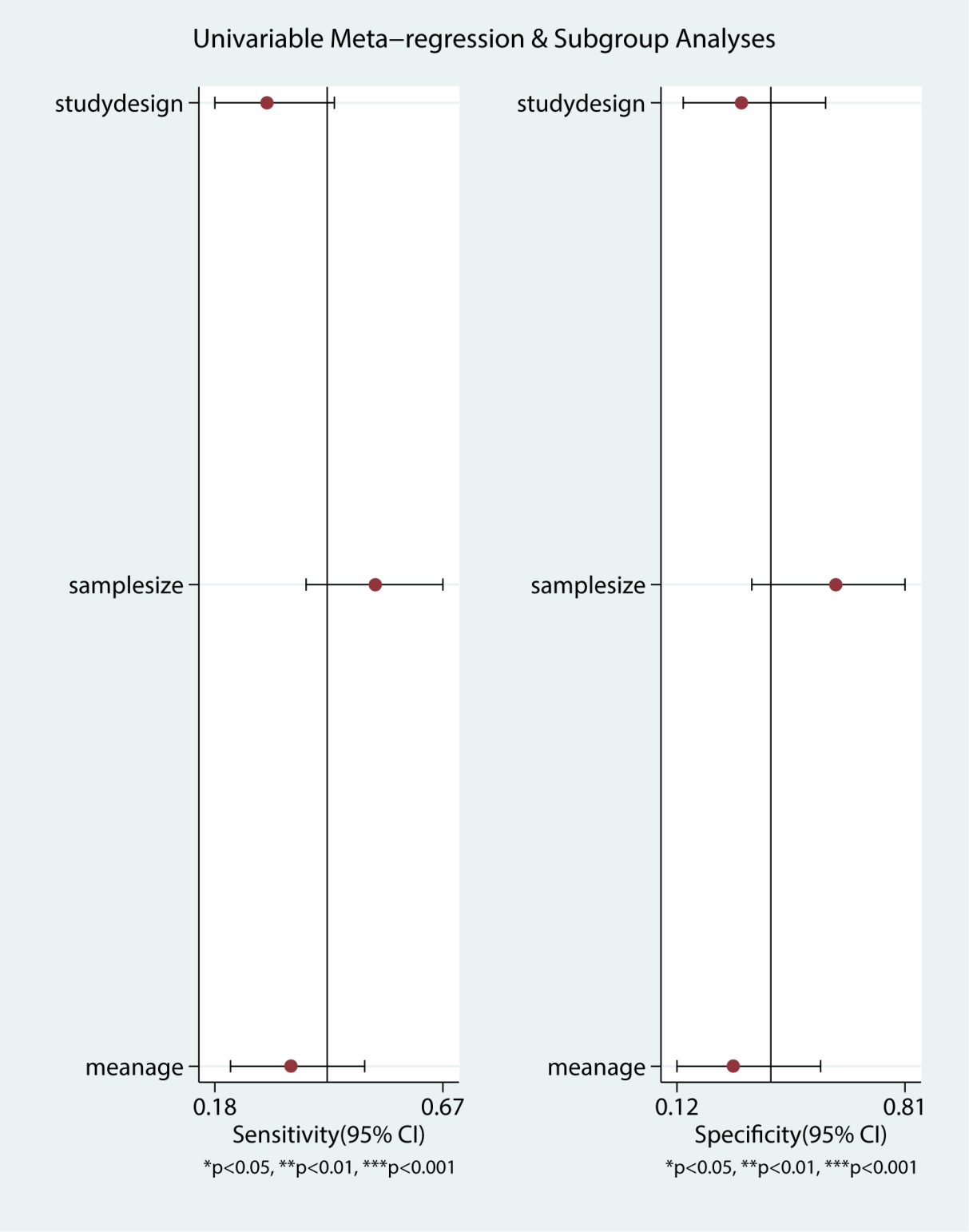


Figure S2. Meta-regression for FDG-PET or FDG-PET/CT based on sensitivity and specificity
