# Supplementary material for: MRI VS. FDG-PET for diagnosis of response to neoadjuvant therapy in patients with locally advanced rectal cancer
Source: Front Oncol. 2023 Jan 18;13:1031581. doi: 10.3389/fonc.2023.1031581 (PMC9890074; doi:10.3389/fonc.2023.1031581)
Supplement: Supplementary file 2 [file Table_1.pdf]

Supplementary table 1. The baseline characteristics of included studies

Study and publication year	Country	Study design	Sample size	Age (years)	No of men and women	Preoperative regimen	Diagnostic tool	Responders and non-responders	Study quality
Amthauer 2004 [28]	Germany	Pro	20	53.1	14/6	RC	FDG-PET	Res: 13; NR: 7	Moderate
Capirci 2004 [29]	Italy	Retro	81	63.9	53/28	RC	FDG-PET	Res: 49; NR: 32	Moderate
Denecke 2005 [30]	Germany	Pro	23	53.0	16/7	RC	FDG-PET	Res: 13; NR: 10	Moderate
Cascini 2006 [31]	Italy	Pro	33	58.0	20/13	RC	FDG-PET	Res: 18; NR: 15	Moderate
Melton 2007 [32]	USA	Retro	21	61.0	13/8	RC	FDG-PET/CT	Res: 14; NR: 7	Moderate
Kristiansen 2008 [33]	Denmark	Retro	30	63.0	16/14	RC	FDG-PET/CT	Res: 14; NR: 16	Moderate
Capirci 2009 [34]	Italy	Pro	81	58.0	58/23	RC	FDG-PET/CT	Res: 40; NR: 41	High
Rosenberg 2009 [35]	Germany	Pro	30	61.0	20/10	RC	FDG-PET/CT	Res: 19; NR: 10	Moderate
Palma 2010 [36]	Spain	Pro	50	60.0	37/13	RC	FDG-PET/CT	Res: 20; NR: 30	Moderate
Lambrecht 2010 [37]	Belgium	Pro	22	59.8	17/5	RC	FDG-PET/CT	Res: 6; NR: 16	Moderate
Martoni 2011 [38]	Italy	Pro	80	65.0	55/25	RC	FDG-PET/CT	CR: 16; IR: 20; NR: 48	High
Hur 2011 [39]	Korea	Pro	37	59.0	25/12	RC	FDG-PET	Res: 25; NR: 12	Moderate
Yoon 2011 [40]	Korea	Pro	72	66.0	56/16	RC	FDG-PET/CT	Res: 43; NR: 29	Moderate

Kim 2011 [41]	Korea	Pro	34	58.1	24/10	RC	MRI	Res: 16; NR: 18	Moderate
Kim 2011 [42]	Korea	Retro	76	60.0	49/27	RC	MRI	CR: 11, nearly CR: 14; MR: 51	Moderate
Herrmann 2011 [43]	Germany	Pro	28	61.0	20/8	RC	FDG-PET/CT	Res: 20; NR: 8	Moderate
Guerra 2011 [44]	Italy	Pro	31	67.0	23/8	RC	FDG-PET/CT	Res: 22; NR: 9	Moderate
Everaert 2011 [45]	Belgium	Pro	45	65.4	34/11	R	FDG-PET	Res: 20; NR: 25	Moderate
Curvo-Semedo 2011 [46]	Netherlands	Retro	50	71.5	36/14	RC	MRI	CR: 14; IR: 36	Moderate
Song 2012 [47]	Korea	Retro	50	56.0	39/11	RC	MRI; FDG-PET/CT	CR: 6; near CR: 13; MR: 31	Moderate
Ippolito 2012 [48]	Italy	Pro	30	66.0	21/9	RC	MRI; FDG-PET/CT	Res: 21; NR: 9	Moderate
Perez 2012 [49]	Brazil	Pro	99	60.3	47/52	RC	FDG-PET/CT	CR: 18; IR: 81	High
Lambrecht 2012 [50]	Belgium	Retro	20	60.0	16/4	RC	MRI	CR: 6; NR: 14	Moderate
Jung 2012 [51]	Korea	Retro	35	62.0	29/6	RC	MRI	Res: 23; NR: 12	Moderate
Janssen 2012 [52]	Netherlands	Pro	51	NA	NA	RC	FDG-PET/CT	Res: 17; NR: 29	Moderate
Huh 2012 [53]	Korea	Pro	50	64.0	38/12	RC	FDG-PET/CT	Res: 32; NR: 18	Moderate
Chennupati 2012 [54]	USA	Retro	35	NA	NA	RC	FDG-PET/CT	CR: 6; near-CR: 8; NR: 21	Moderate

Barbaro 2012 [55]	Italy	Pro	62	64.0	43/19	RC	MRI	Res: 37; NR: 25	High
Guillem 2013 [56]	USA	Pro	121	60.0	76/45	RC	FDG-PET	CR: 26; IR: 95	High
Hatt 2013 [57]	France	Retro	28	67.0	18/10	RC	FDG-PET	Res: 12; NR: 16	Moderate
Murcia Duréndez 2013 [58]	Spain	Pro	41	66.0	25/16	RC	FDG-PET/CT	Res: 14; NR: 27	Moderate
Calvo 2013 [59]	Spain	Pro	38	62.0	27/11	RC	FDG-PET/CT	Res: 19; NR: 19	Moderate
Sun 2013 [60]	China	Pro	53	53.0	44/9	RC	FDG-PET/CT	Res: 21; NR: 32	Moderate
Genovesi 2013 [61]	Italy	Pro	28	68.3	17/11	RC	MRI	Res: 10; NR: 18	Moderate
Park 2014 [62]	Korea	Retro	88	59.2	64/24	RC	FDG-PET/CT	CR: 17; non-CR: 71	Moderate
Niccoli-Asabella 2014 [63]	Italy	Pro	56	62.3	38/18	RC	FDG-PET/CT	Res: 23; NR: 33	High
Cai 2014 [64]	China	Retro	65	56.0	52/13	RC	MRI	Res: 43; NR: 22	Moderate
Aiba 2014 [65]	Japan	Retro	40	56.0	32/8	C	MRI; FDG-PET/CT	Res: 16; NR: 24	High
Doi 2015 [66]	Japan	Pro	16	62.5	13/3	RC	MRI	Res: 9; NR: 7	Moderate
Blažić 2015 [67]	Serbia	Pro	58	61.3	38/20	RC	MRI	Res: 19; NR: 39	Moderate
Martens 2015 [68]	Netherlands	Retro	146	64.6	90/56	RC	MRI	CR: 29; non-CR: 117	High
Petrillo 2015 [69]	Italy	Pro	29	62.0	NA	RC	MRI	Res: 14; NR: 15	Moderate
Choi 2015 [70]	Korea	Retro	86	64.3	58/28	RC	MRI	CR: 16; non-CR: 70	High
Leccisotti 2015 [71]	Italy	Pro	126	65.0	79/47	RC	FDG-PET/CT	CR: 31; non-CR: 95	High

Tong 2015 [72]	China	Pro	38	52.0	25/13	RC	MRI	CR: 12; non-CR: 26	Moderate
Martens 2015 [73]	Netherlands	Pro	30	66.0	23/7	RC	MRI	Res: 13; NR: 17	Moderate
Altini 2015 [74]	Italy	Pro	68	63.0	41/27	RC	FDG-PET/CT	Res: 25; NR: 43	Moderate
Lambregts 2015 [75]	Netherlands	Retro	112	67.0	76/36	RC	MRI	CR: 20; non-CR: 92	Moderate
Koo 2016 [76]	Korea	Retro	103	66.0	78/25	RC	FDG-PET/CT	CR: 22; non-CR: 81	High
Travaini 2016 [77]	Italy	Pro	41	61.0	26/15	RC	FDG-PET/CT	Res: 23; NR: 18	Moderate
Li 2016 [78]	China	Pro	64	53.0	49/15	RC	FDG-PET/CT	Res: 31; NR: 33	Moderate
De Cecco 2016 [79]	Italy	Pro	12	63.2	4/8	RC	MRI	Res: 9; NR: 3	Moderate
Chen 2016 [80]	China	Retro	100	55.0	68/32	RC	MRI	CR: 50; non-CR: 50	Moderate
Iannicelli 2016 [81]	Italy	Pro	34	65.0	19/15	RC	MRI	Res: 11; NR: 23	Moderate
Sathyakumar 2016 [82]	India	Pro	64	49.5	48/16	RC	MRI	CR: 11; non-CR: 53	High
Jacobs 2016 [83]	Netherlands	Pro	22	62.9	16/6	RC	MRI	Res: 9; NR: 13	Moderate
Petrillo 2017 [84]	Italy	Retro	35	67.0	27/8	R	MRI	Res: 16; NR: 19	Moderate
Bassaneze 2017 [85]	Brazil	Retro	33	59.6	18/15	RC	MRI	CR: 7; non-CR: 26	Moderate
De Felice 2017 [86]	Italy	Pro	37	62.0	28/9	RC	MRI	CR: 11; non-CR: 26	Moderate
Zhu 2017 [87]	China	Pro	98	57.5	64/34	RC	MRI	CR: 19; non-CR: 79	High
Yu 2017 [88]	China	Retro	41	NA	25/16	RC	MRI	Res: 17; NR: 24	Moderate

Petrillo 2018 [89]	Italy	Pro	88	66.0	62/26	RC	MRI	Res: 52; NR: 36	Moderate
Fusco 2018 [90]	Italy	Retro	34	67.0	26/8	R	MRI	Res: 15; NR: 19	Moderate
Murata 2018 [91]	Japan	Retro	36	66.0	27/9	RC	MRI; FDG-PET/CT	CR: 10; non-CR: 26	Moderate
Liu 2018 [92]	China	Pro	124	59.0	75/49	RC	MRI	CR: 20; non-CR: 104	Moderate
Aker 2018 [93]	UK	Retro	103	NA	NA	RC	MRI	CR: 20; non-CR: 83	Moderate
Horvat 2018 [94]	Brazil	Retro	114	55.0	67/47	RC	MRI	CR: 21; non-CR: 93	High
Pizzi 2018 [95]	Italy	Pro	43	67.4	22/21	RC	MRI	CR: 21; non-CR: 22	High
Nahas 2019 [96]	Brazil	Retro	95	62.9	58/37	RC	MRI	CR: 20; non-CR: 75	Moderate
Giannini 2019 [97]	Italy	Retro	52	68.0	35/17	RC	MRI; FDG-PET	Res: 22; NR: 30	Moderate
Palmisano 2020 [98]	Italy	Pro	43	61.0	27/16	RC	MRI	Res: 33; NR: 10	High
Bae 2020 [99]	Korea	Retro	38	60.0	17/21	RC	MRI	CR: 26; non-CR: 12	Moderate
López-López 2021 [100]	Spain	Pro	68	63.4	36/32	RC	FDG-PET/CT	CR: 15; non-CR: 53	High
Uemura 2021 [101]	Japan	Retro	40	68.5	26/14	RC	MRI	Res: 17; NR: 23	Moderate

*C: chemotherapy; CR: complete responder; IR: incomplete responder; MR: moderate or minimal responder; NR: non-responder; Pro: prospective; R: radiotherapy; RC: radiochemotherapy; Res: responders; Retro: retrospective.
